# Economic evaluation of the national school food standards across secondary schools in the Midlands, UK (the FUEL study): methodological challenges of undertaking health economics research within non-health settings

**DOI:** 10.1186/s12966-025-01840-6

**Published:** 2025-11-12

**Authors:** Irina Pokhilenko, Miranda Pallan, Marie Murphy, Peymane Adab, Breanna Morrison, Alice Sitch, Ashley Adamson, Suzanne Bartington, Rhona Duff, Tania Griffin, Kiya Hurley, Emma Lancashire, Louise McLeman, Sandra Passmore, Maisie Rowland, Vahid Ravaghi, Suzanne Spence, Emma Frew

**Affiliations:** 1 Centre for Economics of Obesity, Department of Applied Health Research, College of Medicine and Health, University of Birmingham, Birmingham, UK; 2 Department of Applied Health Research, College of Medicine and Health, University of Birmingham, Birmingham, UK; 3 NIHR Birmingham Biomedical Research Centre, Institute of Translational Medicine, University Hospitals Birmingham NHS Foundation Trust and University of Birmingham, Birmingham, UK; 4 Population Health Sciences Institute, Faculty of Medical Sciences, Newcastle University, Newcastle, UK; 5 Department for Health, University of Bath, Bath, UK; 6 Services for Education, Birmingham, UK; 7 The School of Dentistry, University of Birmingham, Birmingham, UK

**Keywords:** Economic evaluation, Costing, School food, Nutritional intake, Health-related quality of life, Educational performance

## Abstract

**Background:**

Economic evaluations of complex public health interventions are becoming increasingly important. This presents health economists with challenges of adapting methodologies originally designed for healthcare to other contexts, such as education. This study presents an economic evaluation of the UK School Food Standards (SFS), with a particular focus on the methodological challenges involved.

**Methods:**

The economic evaluation was conducted alongside an observational study comparing the SFS-mandated secondary schools to non-mandated schools in the Midlands (UK). Costs of food provision and SFS implementation were collected directly from schools and supplemented by secondary data on schools’ catering expenditure. The outcomes included dietary intake, dental health, health-related quality of life (HRQoL), and educational performance, collected from pupils and secondary data. The analysis comprised a micro-costing, cost-consequence, and an exploratory cost-utility analysis, from school and societal perspectives.

**Results:**

Data were collected from 36 schools and 2,543 pupils. We found mandated schools spent less on food provision compared to non-mandated schools, and pupils attending mandated schools had marginally better HRQoL, dental health, and slightly worse nutritional intake. Mandated schools performed worse according to the educational outcomes. There were large amounts of missing cost data despite repeated data collection attempts, and the results of the cost-utility analysis were uncertain.

**Discussion:**

We found no clear evidence on the cost-effectiveness of the SFS in secondary schools, likely due to substantial variation in implementation and compliance across both mandated and non-mandated schools, as well as multiple challenges, including the COVID-19 pandemic, difficulties in collecting cost data from schools, and the complexity of the study context. This study highlights the challenges of primary cost data collection for evaluating complex interventions and the need to balance data accuracy with the resources required. As economic evaluations of school-based interventions become more common, there is a growing need to refine methods for such evaluations.

**Supplementary Information:**

The online version contains supplementary material available at 10.1186/s12966-025-01840-6.

## Introduction

Childhood obesity rates are rising across Europe, with one in three school-aged children now living with overweight or obesity [1]. This is a major societal problem due to the associated co-morbidities [2–4], the negative impact on educational attainment [5, 6], and because early obesity often persists into adulthood, affecting long-term productivity [7] and income [8]. Childhood obesity is also a major driver of health inequalities, with children from disadvantaged backgrounds being at far higher risk, and this pattern is presented across many European countries [9, 10]. Despite multiple government strategy documents such as, for example, the UK Department of Health and Social Care’s policy report ‘Tackling obesity: empowering adults and children to live healthier lives’ [11], calling for a ‘system-wide’ approach across sectors such as transport, environment, workplace, and retail, governments are struggling to slow the rise in population obesity [12, 13].

Good nutrition is crucial for supporting optimal health and well-being, and for preventing obesity during childhood and adolescence. Evidence shows that in England, children’s nutritional intake is suboptimal: they consume excessive amounts of sugar and fat, lower than the recommended amount of fibre, and fewer than recommended portions of fruit and vegetables [14]. Furthermore, approximately half of English adolescents have had experience of dental caries [15]. Poor nutrition and dental health can negatively impact not only children’s overall health and well-being [2, 3], but also their educational attainment [16], through mechanisms such as presenteeism or absenteeism due to health issues or medical appointments. These effects are particularly pronounced among socioeconomically disadvantaged groups [16].

To address childhood obesity, the education sector has been a key focus, leading to the increase in the volume of research within schools to identify cost-effective and sustainable interventions [17]. With children and adolescents spending a significant amount of time at school, the school environment has a substantial impact on their dietary intake and the formation of their dietary behaviours [18, 19]. Therefore, food provision in schools provides an opportunity for policy intervention to promote healthy eating behaviour and ultimately reduce or prevent obesity.

To achieve this, the UK government introduced the School Food Standards (SFS) [20]. The SFS set out the criteria school lunches must meet and are designed to help children develop healthy eating habits and ensure they have sufficient energy and nutrition to participate fully in the school day. All state schools and academies in England are required to comply with the SFS. The SFS legislation was introduced in 2006 and revised in 2015 [21, 22]. However, academies and free schools established between 2010 and 2014 were historically exempt from this mandate and remained exempt following the 2015 revision [23]. These schools were instead encouraged to comply with the SFS [24]. Furthermore, the School Food Plan (SFP) was developed to accompany the SFS and support the implementation of a whole-school approach to healthy eating [25]. The SFP consists of a series of recommendations for action that schools can take to support a healthy eating culture, including actions related to school leadership, the school environment and the curriculum, as well as actions related to the implementation and monitoring of the SFS.

Existing evaluations of the impact of the SFS have shown some improvement in the nutritional intake of primary school pupils [26–28]; however, there is limited evidence on the impact of the SFS in secondary schools and on the implementation of the SFP recommendations. Nicholas et al. (2013) assessed the SFS’s impact across 80 secondary schools. The authors reported increased nutritional quality of school food, reduced availability of confectionery, and small improvements in nutritional intake during lunchtime following the SFS introduction in 2006 [29]. Spence et al. (2014) surveyed pupils in 6 secondary schools in 1999–2000 and 2009–10, and only found limited evidence on the SFS’s impact on the adolescents’ total diet [30]. Furthermore, there is currently no evidence on the SFS’s cost-effectiveness.

The implementation context of the SFS created an opportunity for conducting a quasi-experimental study to investigate the impact of the SFS. Against this backdrop, the Food provision, cUlture and Environment in secondary schooLs (FUEL) study was designed to evaluate the impact of the SFS on secondary pupils’ dietary intake, and to generate evidence on the SFS’s cost-effectiveness, comparing mandated and non-mandated schools located in the Midlands area of the UK [31]. The study found little difference in the extent of compliance with the SFS between mandated and non-mandated schools [32, 33]. Although pupils in mandated schools had lower free sugar intake at lunchtime, the difference compared to non-mandated schools was small. Furthermore, the level of compliance with the SFS showed little association with pupils’ nutritional intake.

This paper presents the economic evaluation conducted alongside the FUEL study. While economic evaluations of school-based interventions are becoming more common [34, 35], adapting methods traditionally used in healthcare to educational settings presents unique challenges. In the paper, we first report on the methods and the results of the economic evaluation. We then discuss the methodological challenges encountered from using health economics methods within a school setting and outline recommendations for future research in this area. The emphasis of the paper is therefore not only on the results of the analysis, but also on the methodological lessons.

## Methods

### FUEL study

The FUEL study was an observational mixed-methods study. It involved collection of data on the SFS and SFP implementation and pupil outcomes in the SFS-mandated and SFS-non-mandated schools. Secondary phase academies and free schools located within 14 Local Authority (LA) areas in the West Midlands and eight LA areas in the East Midlands were recruited. First, all relevant schools were identified and categorised based on the SFS status. Second, stratified sampling based on propensity scores was used to generate a balanced school sample. School characteristics including LA area, number of pupils, proportion of female pupils, proportion of pupils from black and minority ethnic groups, proportion of pupils with English as an additional language, proportion of pupils eligible for free school meals, establishment type, urban/rural, Income Deprivation Affecting Children Index (IDACI), inclusion of a sixth form, selective/non-selective admissions policy, religious affiliation/secular, and number of pupils with special educational needs were used to derive the propensity scores. Propensity scores were then used to stratify the sampling to ascertain comparability across the two school groups. Schools in each sampling group were invited to participate in the study in a random order. All year 7 (11–12 years old), 9 (13 to 14 years old), and 10 (14–15 years old) pupils within the recruited schools were invited to participate. Recruitment took place between November 2019 and April 2022, with a break between March 2020 and May 2021 due to the COVID-19 pandemic. Passive consent and written assent were obtained from parents and pupils, respectively. Schools received £300 and a tailored report detailing the school’s compliance with the SFS and implementation of SFP actions; pupils received £5 as a reward for participating.

The sample size was calculated based on the primary outcome—free sugar intake. Based on a meaningful difference of free sugar intake of 4 g between pupils SFS-mandated vs. SFS-non-mandated schools, a sample of 22 schools and 990 pupils in each group (total schools = 44, total pupils = 1980, average cluster size = 45) was estimated to give over 90% power at the 5% significance level. Following the challenges of the COVID-19 pandemic and with the approval of the Study Steering Committee, the sample size calculation was revised based on the data already collected. We had higher cluster sizes (average pupils = 68 per school) and a higher number of SFS-non-mandated schools, therefore, we estimated that a sample size of 14 schools in the SFS-mandated, and 20 schools in the SFS non-mandated groups would enable us to detect a difference of 4 g in free sugar intake with 87% power at the 5% significance level.

More information on the design of the FUEL study is available in the study protocol [31]. Please refer to Pallan et al. (2024) and the study report for the detailed overview of the results of the primary study highlighting schools’ compliance with the SFS and SFP actions, and the impact of the SFS on pupils’ nutritional intake [32, 33].

### Economic evaluation

The economic analysis included a micro-costing, cost-consequence analysis (CCA), and an exploratory cost-utility analysis (CUA), comparing costs and outcomes associated with the SFS and the SFP between the SFS-mandated and non-mandated secondary schools. The micro-costing adopted a school and a societal perspective, considering costs borne by schools, catering providers, and volunteers. The costs of implementing and delivering the SFS and the SFP, plus the costs of school food provision were calculated for each school using data obtained through a school-reported costing survey (Additional File 1). For the CCA, the costs of implementing and delivering the SFS and the SFP, the costs of school food provision, and selected consequences (i.e. pupils’ outcomes) were summarised in the form of a balance sheet for the two groups of schools being compared. The CCA adopted a school and a societal perspective considering the distribution of costs and consequences borne by schools, catering companies, volunteers, and households. The CUA adopted a school perspective and highlighted the differences in the costs between the two groups of schools being compared offset against the differences in pupils’ Quality-Adjusted Life Years (QALYs).

The time horizon for the analyses was one year. No discount rate was applied. All costs were converted to British Pounds 2021 based on the UK Consumer Price Index as published by the Office for National Statistics [36]. Statistical analyses were conducted using STATA version 17. The health economic analysis plan was developed prior to commencing the analysis and is available upon request from the corresponding author. Full ethical approval for the FUEL study was obtained from the University of Birmingham Ethical Review Committee on the 20th August 2019 (ERN_18-1738). The economic evaluation is reported in line with Consolidated Health Economic Evaluation Reporting Standards (CHEERS) [37]. Please refer to Additional File 3 to view the completed CHEERS checklist for this study.

#### Cost data

##### Primary data collection

The costing survey included questions to capture any one-off, or occasional costs associated with the implementation and delivery of the SFS and the SFP; ongoing annual costs of food provision; and costs associated with other activities to support healthy eating, including growing food, gardening and cooking clubs, cooking lessons, school food governance, and staff training on health and well-being. The survey was developed by the FUEL study team as a paper-based questionnaire and initially tailored to individual schools based on their characteristics. To support this, a key information survey, including tick-boxes on the presence/absence of features of the school food system/environment, was first sent to all participating schools. Responses to this key information survey were then used to tailor the costing survey so that it addressed only those features of the school food system/environment that were ticked off as present, while questions relating to absent features were omitted. Before deployment, the survey was reviewed by two members of the FUEL public advisory group, who were senior school leaders, and edited based on their feedback on the wording and ease of completion. The survey was sent to key contact persons (e.g. school business managers) in participating schools to be completed.

After an initial low response rate and poor completion, the costing survey was shortened to focus on the most relevant cost items and converted to an online format using REDCap in November 2021. Following this revision, it was no longer tailored to individual schools. Instead, it adopted skip logic, where for each feature of the school food environment, respondents were first asked if the feature was present, and if so, to provide information about associated costs. If a feature was absent, they could skip to the next section. The final (online) version of the survey was also reviewed by a public advisor prior to deployment. Both versions of the costing survey are available in Additional Resource 1.

Resource use collected via the school survey was valued in monetary terms using appropriate UK unit costs listed in Table 1 or participant valuations estimated at the time of data collection.

**Table 1:** Unit costs for the valuation of resource use

Item	Unit of measurement	Unit cost	Description	Source
Teaching staff time	Per hour	£20	Gross average annual salary for teachers in England (excluding the London area) divided by 39 weeks, 5 working days and 8 working hours	Teachers’ salary: Department of Education [38] Teachers’ working weeks: NASUWT Teacher’s Union [39]
Catering staff time	Per hour	£11.47 (catering assistants) £19.47 (catering manager, chef)	Local government pay scale for support staff, hourly pay mid-scale of the lower and the higher portions of the scale	National Education Union [40]
Vending machine maintenance	Per week	£46	Weekly average rental	School Food Trust [41]
Water fountain maintenance	Per year	£17	Average cost of maintaining a water fountain	School data: one FUEL school reported spending £50 per year on maintaining 3 water fountains
Volunteer time	Per hour	£8.91	National Living Wage (for 23 and older), the unit cost for an hour of unpaid work	UK Government [42]
Ingredients for cooking classes	Average cost per pupil spent every fourth lesson	£5	£5 was an assumption made by the study team corroborated by publicly available estimates [43]. It was used in the cost calculation, unless an estimate was reported by schools	Islington Council [43]

Costs for pupils and families were collected in the pupil survey (Additional Resource 2). The pupils were asked to indicate how much they typically spent on food on a school day, purchased both at school and at food outlets outside of school. The answer options were given in ranges (e.g. £1 - £2.99). The average spending per pupil per day was calculated by using the midpoint value for each category and summed to calculate the total average spending per pupil.

##### Secondary data

School resource-use data had large amounts of missing data. Therefore, the CCA and CUA analyses were supplemented with secondary data on catering expenditure [44]. All schools in England are required to report expenditure data under pre-determined budget headings and this information was publicly available on an annual basis for both catering staff and catering supplies (including the cost of providing free school meals and milk). However, it did not include expenditures associated with mealtime assistance and midday supervisors, any training for catering staff, and costs related to maintenance and improvement to the canteen. For all schools, the catering expenditure for the year 2018–2019 was used to reflect the pre-pandemic situation. This was with the exception of four schools for which all data were collected during the pandemic (2020–2021 academic year), and therefore the same year was used for the catering expenditure.

#### Outcomes

Outcome measures included: dietary intake, dental health outcomes, educational outcomes, and QALYs. Data on dietary intake, dental health, and QALYs were collected from pupils using study-specific questionnaires (Additional File 2) and a 24-hour dietary recall tool in two sessions approximately 2–4 weeks apart.

##### Dietary outcomes

Dietary intake was measured using Intake24, an online self-reported 24-hour recall tool for recording dietary intake [45]. The Intake24 data was used to estimate outcomes for three time periods – school lunch, school day (i.e. intake during the time spent in school), and whole day (24 h), and included: free sugar intake, percentage of dietary energy intake from free sugar, total energy intake, total fat intake, fibre intake, number of fruit and vegetable (F&V) portions consumed, number of sugar-sweetened beverages (SSBs) consumed, number of sugar and chocolate confectionery items consumed, and number of foods high in fat, sugar and salt (HFSS) consumed. In the CCA, all dietary outcomes presented were for the school day. Additional outcomes related to dietary intake during 24 h and included in the CCA were: >5% of total energy intake from free sugar, consumption of 5 or more portions of F&V, and number of eating/drinking occasions (excluding plain water).

##### Dental health outcomes

Dental health outcomes were measured using validated measures from the national Child Dental Health Survey [15] and included presence of any symptoms indicating dental caries in the past three months, the number of dental caries symptoms in the past three months, and past dental caries treatment.

##### Quality-adjusted life years

QALYs were measured using the Child Health Utility 9-Dimension (CHU-9D) instrument. CHU-9D is a generic preference-based measure of health-related quality of life (HRQoL) suitable for 7 to 17-year olds designed to be self-completed by the child. It consists of a 9-question survey with 5 response levels and a set of preference weights generated using general population values. CHU-9D has previously been used for the measurement of QALYs in general population samples of children and adolescents and has shown to have good psychometric performance [46]. Utility scores were derived from responses to the CHU-9D using preference weights obtained from a sample of the UK adult general population [47].

##### Educational outcomes

Educational outcomes data were collected from publicly available school performance tables [48] for the year 2019 to reflect school performance before the COVID-19 pandemic. Relevant indicators were identified by the FUEL study team in consultation with the Study Steering Committee and school public advisors, and included absenteeism, and secondary educational performance, such as progress 8 score (a measure of the progress children make between the end of primary school and the end of secondary school relative to the national average, based on attainment 8 scores), attainment 8 score (a measure of the average academic performance of a secondary school in eight defined subjects), staying in education or entering employment, and grade 5 or above in English & Maths General Certificate of Secondary Education (GCSEs).

#### Micro-costing

All schools that completed the costing survey were included in the micro-costing analyses. Resource use was summarised for each school separately, multiplied by the relevant unit costs, and summed to calculate the total costs of implementing and delivering the SFS and the SFP. The wider costs of food provision and creating a healthy food culture, including the costs for schools, catering providers, and volunteer time were considered. To calculate the costs of food provision and creating a healthy food culture per pupil, total costs were divided by the number of pupils in the relevant school for the same year as when the cost data were collected. Given that a large proportion of costs related to food provision, school meal uptake was accounted for by also calculating costs per pupil based on the number of pupils reported to have school meals. Where these data were not available on the school level, school meal uptake was estimated from pupil participant data (Intake24). We also calculated the percentage of missing cost data for each school.

Several assumptions had to be made beyond those mentioned in Table 1. To calculate resource use associated with mealtime supervision, we assumed that breakfast lasted fifteen minutes, break lasted fifteen minutes, and lunch lasted one hour. For some schools, data on mealtime duration were available and were used instead. To calculate the cost of classroom cover during staff training, it was assumed that one training session lasted two hours and corresponded to the unit cost of teaching staff time (£20 per hour). The school gardening club sessions were assumed to last one hour with one staff member leading the session.

#### Cost-consequence analysis

The CCA listed the costs of food provision calculated from the micro-costing analysis (both from a school and a societal perspective); schools’ catering expenditure based on secondary data; and costs for pupils/families, including the cost of buying food during the school day and the cost of buying the ingredients for cooking lessons. The following outcomes were included: QALYs; educational outcomes; dietary intake outcomes; and dental health outcomes as described above.

The average values were calculated for the SFS-mandated and SFS-non-mandated schools. For the data collected at the school level, i.e. costs and educational outcomes, the average values were calculated based on the school average values. For the data collected at the individual level, i.e. dietary intake, dental health, and QALYs, the average values for all pupils in each school group were calculated.

#### Exploratory cost-utility analysis

Due to the high proportion of missing data within the costing surveys, secondary catering expenditure data was used for the CUA. Therefore, the analysis adopted a school perspective. All schools, in which pupils provided HRQoL data, were included in the CUA. Two analyses were conducted: the first analysis used catering expenditure per pupil based on the total number of pupils, and the second used catering expenditure per pupil based on school meal uptake.

The difference in catering expenditure per pupil was offset against the differences in average QALYs in the SFS-mandated vs. non-mandated schools to calculate the incremental cost-effectiveness ratio (ICER), using linear regression. Both analyses were adjusted for pupil and school characteristics. The difference in QALYs was adjusted for age (if age was missing, average age for the year group was imputed), Index of Multiple Deprivation (IMD) quintile, sex, ethnicity, and source of school lunch (100% school-provided vs. other). The difference in costs was adjusted for the mode of catering provision (in-house vs. externally contracted), academic year of data collection, IDACI score, percentage of black and ethnic minority pupils, percentage of pupils eligible for free school meals, percentage of pupils whose first language was other than English or Welsh, and academy status.

Non-parametric bootstrapping was performed to simulate 5,000 cost and QALY pairs and generate a cost-effectiveness plane to illustrate the likelihood of the SFS being cost-effective for both analyses separately. We also constructed two cost-effectiveness acceptability curves (CEACs): one using catering expenditure per pupil based on the total number of pupils, and the other using catering expenditure per pupil based on school meal uptake, to estimate the probability of the SFS being cost-effective under different willingness-to-pay thresholds. The commonly applied threshold within a health care setting of £20,000 - £30,000 per QALY was used to judge cost-effectiveness.

## Results

### Descriptive characteristics of the sample

36 schools (out of 482 invited) were recruited to participate. 23 were non-mandated and 13 were mandated to comply with the SFS legislation. We invited 2,575 pupils from year groups 7, 9 and 10, and 2,543 gave their assent with passive parental consent. Of these participants, 865 were in the SFS-mandated, and 1,678 were in the SFS-non-mandated schools. 2,273 pupils provided nutritional intake data, 2,268 provided dental outcome data, and 1,495 provided HRQoL data. The SFS-mandated school group had a higher proportion of participants: from more deprived areas; in receipt of free school meals; and of white ethnicity, and a lower proportion of female participants (Table 2).

**Table 2:** Pupil characteristics

Characteristic	Total *n* = 2,543 *n* (%)	Attending SFS-mandated schools *n* = 865 *n* (%)	Attending SFS-non-mandated schools *n* = 1,678 *n* (%)
Year group			
7	859 (33.78)	304 (35.14)	555 (33.08)
9	850 (33.43)	279 (32.25)	571 (34.03)
10	834 (32.80)	282 (32.60)	552 (32.90)
Age (years); mean (SD)	13.63 (1.29)	13.64 (1.33)	13.63 (1.27)
Missing	68	24	44
IMD quintile group			
1 (highest deprivation)	585 (25.50)	259 (33.86)	326 (21.32)
2	389 (16.96)	160 (20.92)	229 (14.98)
3	460 (20.05)	168 (21.96)	292 (19.10)
4	425 (18.53)	91 (11.90)	334 (21.84)
5 (lowest deprivation)	435 (18.96)	87 (11.37)	348 (22.76)
Missing	249	100	149
Sex			
Female	1,377 (54.15)	427 (49.36)	950 (56.62)
Male	1,061 (41.72)	397 (45.90)	664 (39.57)
Other/unknown	105 (4.13)	41 (4.74)	64 (3.81)
Ethnicity			
White	1,762 (69.29)	638 (73.76)	1,124 (66.98)
Asian/Asian British	385 (15.14)	94 (10.87)	291 (17.34)
Black/African/Caribbean/Black British	144 (5.66)	42 (4.86)	102 (6.08)
Mixed/Multiple	142 (5.58)	55 (6.36)	87 (5.18)
Other ethnic group/unknown	110 (4.33)	36 (4.16)	74 (4.41)
Free School Meals			
Yes	193 (12.71)	87 (17.94)	106 (10.26)
No	1,248 (82.21)	367 (75.67)	881 (85.29)
Pupil did not know	77 (5.07)	31 (6.39)	46 (4.45)
Missing	1,011	380	645
Consuming a school-provided lunch			
Never	410 (26.54)	105 (25.86)	305 (26.78)
Less than once a week	150 (9.71)	29 (7.14)	121 (10.62)
1–2 times a week	227 (14.69)	51 (12.56)	176 (15.45)
3–4 times a week	194 (12.56)	55 (13.55)	139 (12.20)
Every school day	564 (36.50)	166 (40.89)	398 (34.94)
Missing	970	459	539

*SFS *School Food Standards, *SD * Standard Deviation, *IMD * Index of Multiple Deprivation

The results of the observational study indicate that the degree of compliance with the SFS was similar in mandated (63.3%) and non-mandated schools (64.5%). Compliance was highest for the SFS applied to lunchtime and lowest for those applying across the whole school day. Compliance was also lower for standards restricting high fat, sugar and energy-dense items than for standards aiming to increase dietary variety. Pupils in mandated schools had a lower free sugar intake during lunchtime. Across all schools there were few significant associations between the degree of SFS compliance and pupil nutritional intake. Please refer to Pallan et al. (2024) for further information [33].

### Micro-costing

22 schools completed the costing survey and were included in the micro-costing analysis. Out of these, 6 were SFS-mandated, and 16 were non-mandated. 13 schools had external catering and 9 schools had in-house catering.

The annual ongoing costs associated with SFS and SFP, and of food provision for schools ranged from £8,500 to £861,950, with a mean of £182,732. The equivalent per-pupil cost ranged from £10 to £869, mean £173 (based on the total number of pupils); and from £16 to £2,311, mean £388 (based on the number of pupils taking school meals). Annual ongoing societal costs of food provision included costs to schools, catering providers and volunteer time and ranged from £8,500 to £974,563 with a mean of £207,094. Annual ongoing societal costs per pupil ranged from £9 to £982, mean £195 (based on the total number of pupils), and from £16 to £2,613, mean £426 (based on the number of pupils taking school meals). Eleven schools did not report any wider costs in addition to the costs borne by the school, hence for these schools the total estimate from the societal perspective was the same as from the school perspective.

Staff costs, including catering staff, teaching staff involved in meal supervision, and teaching staff involved in delivering cooking lessons, were the three largest cost categories; they were also in the top five of the best completed categories with the least amount of missing data. Catering staff costs ranged from £37,166 to £191,100, with a mean of £108,719. Teaching staff costs ranged from £13,974 to £191,100 with a mean of £72,488.

In general, the surveys were not well-completed with the average proportion of missingness of 21% for paper-based surveys and 39% for online surveys (range 0%−89%). The summary of micro-costing is presented in Table 3 and the full overview is presented in Additional File 4. Please note that in the supplementary material the results are presented separately for the schools that completed the paper-based version (*n* = 8) and the online version (*n* = 14) because of the differences in the survey format.

**Table 3:** Micro-costing of school food provision

School ID	Catering provision	% of pupils receiving free school meals	Annual ongoing school costs^a^	School cost per student^a^	Cost per student based on school meal uptake^a^	Annual ongoing societal costs^a, b^	Societal cost per student^a, b^	Societal cost per student based on school meal uptake^a, b^	Average annual spending on cooking ingredients per pupil^a^	% of missing data (ongoing costs only)
SFS-mandated										
ID11	In-house	15	67,851	86	209	67,851	86	209	107	10
ID27	In-house	20	256,744	221	535	256,744	221	535	38	23
ID26	External	21	191,100	172	172	191,100	172	172	ND	0
ID32	External	22	122,400	104	199	220,680	187	359	ND	50
ID36	External	31	M	M	M	191,100	166	256	ND	50
ID44	External	18	64,400	80	167	135,380	169	352	ND	29
SFS-non-mandated										
ID17	In-house	6	222,709	126	187	222,709	126	187	15	11
ID24	In-house	16	176,312	152	303	176,363	152	304	23	18
ID13	In-house	6	212,850	242	520	212,850	242	520	ND	0
ID31	In-house	15	434,460	355	844	434,460	355	844	ND	60
ID33	In-house	10	186,359	195	553	186,359	195	553	ND	0
ID42	In-house	32	8,500	10	16	8,500	10	16	ND	67
ID45	In-house	14	105,740	68	169	105,740	68	169	ND	80
ID01	External	18	231,628	239	328	233,422	241	331	M	67
ID07	External	3	91,394	149	408	92,522	151	413	NA	0
ID22	External	9	115,105	142	237	142,403	176	293	39	14
ID30	External	19	151,389	120	410	151,389	120	410	38	23
ID25	External	13	M	M	M	M	M	M	ND	89
ID29	External	13	70,600	48	63	193,450	131	173	ND	50
ID38	External	49	861,950	867	2,311	974,563	982	2,613	ND	0
ID39	External	3	28,700	19	30	28,700	19	30	ND	30
ID43	External	29	54,440	55	97	122,690	125	218	ND	38
Mean			182,732	173	388	207,094	195	426	43	32

School Food Standards (*SFS*), School Food Plan (*SFP*), Not displayed (*ND*), Missing (*M*), Not applicable (*NA*)

^a^All costs are reported in British Pounds for the year 2021; ^b^Societal costs include costs to schools, catering providers, and volunteers

### Cost-consequence analysis

36 schools (13 mandated and 23 non-mandated) and 2,543 pupils were included in the CCA. The higher number of schools included in the CCA compared to the micro-costing is due to the fact that the CCA also included those schools that did not return the costing survey, but we were still able to retrieve their catering expenditure data. Compared with non-mandated schools, SFS-mandated schools had lower IDACI scores, a higher proportion of pupils eligible for free school meals, lower proportion of pupils from black and ethnic minority groups, lower proportion of pupils whose first language is known or believed to be other than English, and a slightly lower percentage of pupils with special educational needs. The summary of the CCA is presented in Table 4 and the full overview is available in Additional File 5.

**Table 4:** Cost-consequence analysis of the school food standards

School information				Costs				HRQoL	Educational outcomes						Nutritional intake (during school day)						Dental outcomes
School ID	Mode of catering provision	School IDACI	%FSM	School spending on catering (£2021)^a^	School spending on catering per pupil (£2021)^a^	School spending on catering per number of pupils having school meals (£2021)^a^	Average daily spending on food while at school (£2021)^a^	QALYs	Overall absence (%)	Persistent absence (%)	Progress 8 score	Attainment 8 score	Grade 5 or above in English & maths GCSEs (%)	Staying in education or entering employment (%)	Free sugar (g)	%TEI from free sugar	TEI (kcal)	Fat (g)	F&V portions	Fibre (g)	Average number of caries symptoms
SFS-mandated schools (*n* = 13)																					
ID03	In-house	0.12	20	190,486	148	222	3.26	0.8311	5	14	−0.05	46	31	98	22.0	9.9	744	28.7	0.8	7.2	2.1
ID08	External	0.12	25	67,291	79	190	NA	NA	7	16	−0.46	39	26	NA	32.1	17.9	721	30.5	0.9	5.9	2.3
ID11	In-house	0.07	15	56,939	70	175	3.13	0.8438	6	13	−0.43	42	32	90	29.6	14.1	738	29.0	0.8	6.0	1.8
ID12	External	0.12	14	24,846	43	95	2.45	0.7654	5	12	0.57	46	39	95	17.2	10.6	691	27.2	1.2	7.1	1.6
ID16	External	0.20	44	243,136	315	494	NA	NA	5	11	−0.06	46	45	96	36.3	14.6	777	31.2	0.6	5.6	2.4
ID18	In-house	0.22	22	140,794	117	229	NA	NA	5	13	0.23	48	39	95	22.6	12.2	643	26.6	0.5	5.3	2.0
ID26	External	0.23	21	401,000	361	361	2.60	0.8562	2	2	NA	NA	NA	NA	20.6	9.3	583	23.9	0.7	6.1	1.5
ID27	In-house	0.25	20	115,948	104	242	3.60	0.8340	6	20	−0.23	46	45	92	41.8	19.1	753	28.8	0.7	5.6	2.1
ID32	External	0.09	22	104,560	93	170	4.42	0.8321	6	16	NA	NA	NA	NA	23.8	15.6	634	25.8	1.0	5.1	1.8
ID36	External	0.35	31	54,868	48	74	2.93	0.8314	8	25	−0.45	33	22	NA	37.7	17.5	781	29.0	1.1	6.5	1.8
ID41	In-house	0.06	23	236,037	245	387	3.48	0.8297	7	17	−0.01	45	41	NA	25.4	13.9	633	26.8	0.9	5.3	1.7
ID44	External	0.18	18	-	-	-	3.99	0.7971	7	20	−0.39	40	23	NA	30.4	17.5	677	28.2	0.7	5.1	1.7
ID46	External	0.26	20	35,199	42	74	NA	NA	6	15	0.47	48	56	92	32.0	13.9	805	32.7	0.8	7.2	2.1
Mean for SFS-mandated schools				128,547	128	209	3.32	0.8302	6	15	−0.07	43	36	94	28.0	14.1	701	28.1	0.8	6.0	1.9
SFS-non-mandated schools (*n* = 23)																					
ID01	External	0.03	18	133,547	145	189	3.90	0.7998	4	8	0.74	53	49	100	26.4	11.9	795	33.0	0.8	7.0	2.1
ID02	External	0.29	20	314,716	284	580	3.37	0.8565	7	17	−0.63	41	34	94	43.1	18.6	849	34.6	1.4	7.1	1.6
ID05	In-house	0.21	20	77,644	92	318	NA	NA	7	23	−0.44	43	30	91	37.1	17.1	936	39.3	1.1	7.1	1.8
ID06	In-house	0.58	62	181,169	237	440	2.56	0.8480	5	15	−0.08	43	37	91	28.3	16.0	672	27.4	0.7	5.4	1.9
ID07	External	0.01	3	12,000	20	54	2.87	0.7891	3	6	0.74	78	100	100	23.3	10.8	781	33.4	1.6	8.1	2.0
ID10	External	0.17	35	130,442	144	279	3.24	0.7980	6	17	0	46	42	88	30.4	16.0	742	30.3	0.7	5.7	2.1
ID13	In-house	0.10	6	237,072	273	580	2.97	0.8356	4	8	0.61	73	98	98	22.5	12.3	641	24.1	1.6	6.8	2.0
ID14	External	0.05	7	43,481	48	92	2.50	0.8833	6	15	−0.1	47	40	95	24.0	12.6	745	30.7	1.2	6.7	2.0
ID17	In-house	0.02	6	28,987	17	24	3.43	0.8284	4	7	0.45	59	67	97	22.6	10.8	685	27.3	1.2	6.3	2.1
ID22	External	0.08	9	4,000	5	8	4.14	0.8341	4	8	0.23	50	47	94	24.0	14.5	709	29.1	1.0	6.3	2.2
ID24	In-house	0.13	16	235,000	202	404	4.04	0.8545	6	15	−0.13	45	31	96	26.5	17.4	679	28.7	0.7	5.3	2.0
ID25	External	0.13	13	55,904	37	106	3.59	0.8476	5	11	−0.1	47	40	97	26.9	13.1	680	28.6	1.1	5.5	1.9
ID29	External	0.06	13	-	-	-	4.14	0.8324	5	8	0.2	51	49	97	17.6	12.1	522	21.7	0.6	4.2	1.9
ID30	External	0.13	19	46,586	48	126	3.48	0.8292	6	15	0.07	49	49	89	28.9	15.3	769	31.0	0.9	6.3	2.0
ID31	In-house	0.06	15	97,314	95	189	3.45	0.8458	5	11	0.27	52	56	94	28.2	11.3	846	35.1	1.0	6.4	1.9
ID33	In-house	0.23	10	153,217	159	455	3.23	0.7963	5	9	0.27	52	65	NA	22.7	14.3	715	28.6	1.2	6.2	1.9
ID35	In-house	0.03	9	386,148	257	389	3.54	0.8429	5	10	−0.13	47	44	94	22.8	14.3	585	23.4	0.9	5.2	1.8
ID37	External	0.08	9	311,610	387	840	4.74	0.8383	5	8	0.12	54	61	99	31.0	15.5	764	30.3	1.4	6.6	1.9
ID38	External	0.14	49	179,098	192	480	3.90	0.8167	6	16	0.51	44	28	93	26.3	17.2	659	24.3	1.1	5.2	2.2
ID39	External	0.16	3	-	-	-	3.12	0.8381	4	6	0.56	61	74	98	17.7	9.7	647	25.9	1.2	5.8	1.8
ID42	In-house	0.18	32	159,429	212	302	3.65	0.7988	4	5	0.4	45	31	87	28.5	14.0	714	29.2	0.8	5.7	1.9
ID43	External	0.06	29	45,551	61	81	2.42	0.8343	6	15	0.16	45	36	99	21.1	12.2	685	2.2	1.2	6.6	2.2
ID45	In-house	0.07	14	243,284	155	388	3.99	0.7999	5	14	−0.35	44	37	94	21.2	13.1	571	2.2	0.8	4.9	2.2
Mean for SFS-non-mandated schools				133,748	133	275	3.47	0.8274	5	12	0.15	51	50	95	26.2	13.9	715	29.0	1.1	6.1	2.0

*HRQoL* Health-Related Quality of Life, *%FSM * % pupils eligible for Free School Meals, *IDACI * Income Deprivation Affecting Children Index (the proportion of children aged 0–15 years living in income deprived families, measured for the Lower-layer Super Output Area (*LSOA*) in which the school is situated), *QALY * Quality Adjusted Life Years, *SFS * School Food Standards, *TEI * Total Energy Intake, *F&V * Fruit & Vegetable, *NA * Not Available

^a^All costs are reported in British Pounds for the year 2021

#### Costs

The average annual school costs associated with SFS and SFP and of food provision in the SFS-mandated schools were £140,499 compared with £196,809 in the non-mandated schools. The average annual school costs per pupil (based on total number of pupils) were £133 in the SFS-mandated schools vs. £186 in the non-mandated schools. The average annual school costs per pupil based on pupils taking up school meals were £256 in the SFS-mandated schools vs. £432 in the non-mandated schools.

The societal costs were £177,143 in the SFS-mandated schools, and £219,075 in the SFS-non-mandated schools. The societal costs per pupil (based on total number of pupils) were £167 in the SFS-mandated schools and £206 in the SFS-non-mandated schools, and £314 in the SFS-mandated schools and £471 in the SFS-non-mandated schools based on pupils taking up school meals. The percentage of missing data in the SFS-mandated schools was lower compared with the SFS-non-mandated schools (26.9% vs. 34.1%).

The cost differences between mandated and non-mandated schools were corroborated with the public data on catering expenditure. The average annual spending on catering was £128,546 in the SFS-mandated schools and £133,748 in the SFS-non-mandated schools. The average annual spending on catering per pupil (based on the total number of pupils) was £128 in the SFS-mandated schools vs. £133 in the non-mandated schools, and £209 in the SFS-mandated schools vs. £275 in the non-mandated schools based on pupils taking up school meals.

Pupils in the SFS-mandated schools reported spending slightly less on food while at school compared with the pupils in the SFS-non-mandated schools; £3.32 vs. £3.47 per day. Pupils’ annual spending on ingredients for cooking lessons was higher in the SFS-mandated schools compared with the pupils in the SFS-non-mandated schools; £64 vs. £39 per pupil.

#### Outcomes

Pupils in the SFS-mandated schools reported slightly higher CHU-9D utility values compared with the pupils in the SFS-non-mandated schools: 0.8302 vs. 0.8274. SFS-mandated schools had a higher percentage of missing CHU-9D data, with the data available for 9 out of 13 schools (*n* = 390 pupils). In the non-mandated group, the CHU-9D data were available for 22 out of 23 schools (*n* = 1,105 pupils). Non-mandated schools consistently performed better compared with the SFS-mandated schools according to all selected educational outcomes.

Pupils in the SFS-mandated schools had higher sugar intake, higher consumption of SSBs and sugar and confectionery items, and lower consumption of HFSS items. Pupils in the non-mandated schools had a slightly higher total energy intake, fat intake, fibre intake, a higher consumption of F&V, and a higher number of eating and drinking occasions. Furthermore, a higher proportion of pupils in the non-mandated schools consumed 5 or more F&V portions during the whole day.

In terms of dental health, pupils in the SFS-mandated schools had a lower presence of caries, a lower average number of caries symptoms. Furthermore, a higher proportion of pupils in the SFS-mandated schools reported having a caries treatment in the past.

### Exploratory cost-utility analysis

Pupils from 31 schools, for which both public catering expenditure data and QALYs were available, were included in the CUA (Table 5). For the first analysis, the difference in the average catering expenditure per pupil based on the total number of pupils (SFS vs. non SFS schools), -£5.69 (i.e. £5.69 less in the SFS compared to non-SFS group), was offset against the difference in QALYs, 0.003. After adjustment for covariates, the difference in the cost was -£45.61 and the difference in QALYs was 0.001, indicating that the SFS-mandated schools were slightly more favourable than the SFS-non-mandated schools as they spent less on catering and the pupils reported slightly higher HRQoL.

**Table 5:** Exploratory cost-utility analysis comparing SFS-mandated school to SFS-non-mandated schools

		Catering expenditure per pupil (based on the total number of pupils)		Catering expenditure per pupil (based on school meal uptake)	
		Costs	QALYs	Costs	QALYs
School group	SFS-mandated	133.86 (SD: 109.07)	0.830 (SD: 0.125)	214.45 (SD: 137.02)	0.830 (SD: 0.125)
	SFS-non-mandated	139.55 (SD: 107.41)	0.827 (SD: 0.122)	286.45 (SD: 137.02)	0.827 (SD: 0.122)
Unadjusted difference		−5.69, 95% CI: −14.68–3	0.003, 95% CI: −0.011–0.018	−72.15, 95% CI: −85.97 – −57.91	0.003, 95% CI: −0.011–0.018
Adjusted difference		−45.61, 95% CI: −54.75 - −36.09	0.001, 95% CI: −0.015–0.016	−129.37, 95% CI: −145.34 - −111.54	0.001, 95% CI: −0.015–0.016

*SFS* School Food Standards, *QALY * Quality-Adjusted Life Year, *SD * Standard deviation, *CI * Confidence interval

**Fig. 1:**
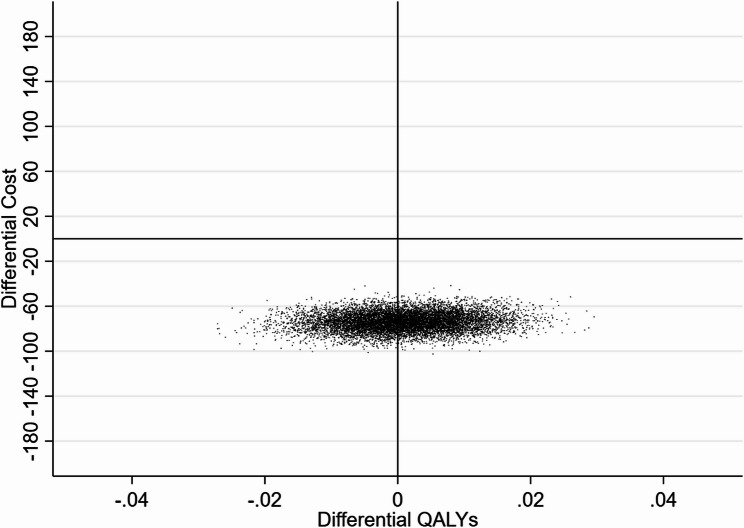
Results of non-parametric bootstrapping comparing SFS with non SFS schools based on the total pupil numbers

The results of the bootstrapping are presented in Fig. 1. All simulated cost and QALY pairs were located in the south of the cost-effectiveness plane with substantial uncertainty in the QALY values.

**Fig. 2:**
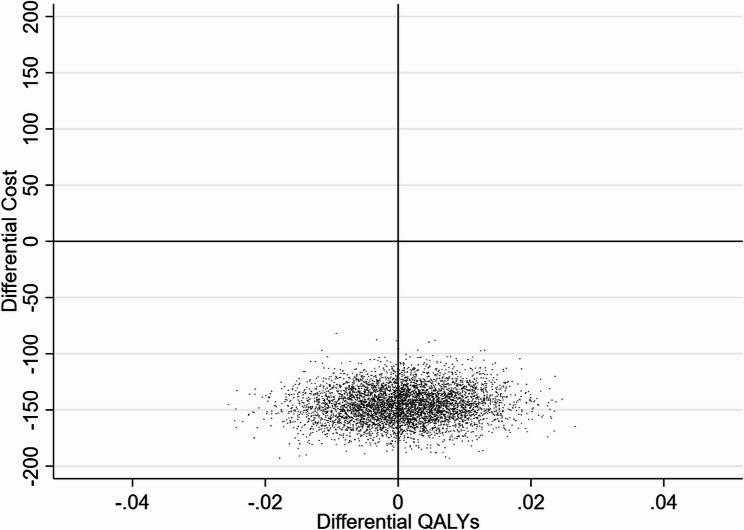
Results of non-parametric bootstrapping comparing SFS with non SFS schools based on the school meal uptake

The second analysis (using only pupils taking school meals) showed similar results and a greater difference in the costs between the two school groups (Table 5). After adjustment for covariates, the difference in costs between SFS and non-SFS schools was -£129.37. Fig. 2 shows the results of the bootstrapping. Overall, in both analyses the SFS-mandated schools spent less on catering compared to the SFS-non-mandated schools, and there were no significant differences in QALYs across the pupils.

**Fig. 3:**
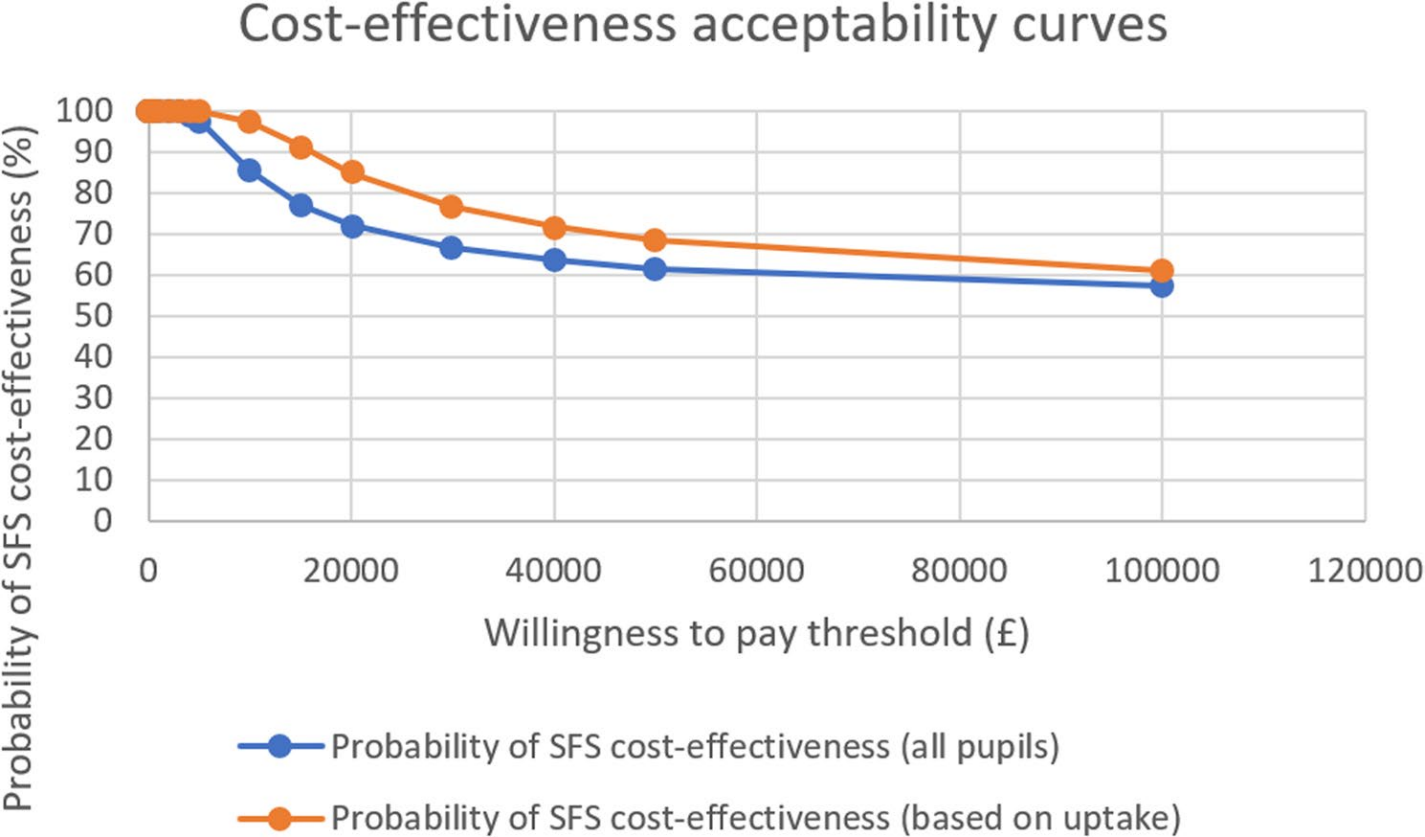
Cost-effectiveness acceptability curves demonstrating the probability of the School Food Standards being cost-effective at different willingness-to-pay thresholds

In Fig. 3, we present CEACs, derived from the joint distribution of costs and effects using non-parametric bootstrapping, to show the probability of the SFS being cost-effective across a range of willingness-to-pay thresholds. Both CEACs demonstrate that the SFS intervention has a high probability of being cost-effective across all willingness-to-pay thresholds examined. For the analysis based on all pupils, the probability of cost-effectiveness remains at 100% for willingness-to-pay thresholds up to £2,000, then gradually decreases to 72% at £20,000, 67% at £30,000, and 57% at £100,000. For the analysis based on pupils taking school meals, the intervention shows higher cost-effectiveness, maintaining 100% probability up to £5,000, decreasing to 85% at £20,000, and remaining above 60% even at the highest applied threshold of £100,000.

## Discussion

To our knowledge, this is the first study to evaluate the cost-effectiveness of the SFS and SFP in UK secondary schools, comparing the schools mandated to adhere to the SFS with those that are not. We hypothesised that pupils in mandated schools would have better dietary intake, HRQoL, and educational outcomes, as compliance with the SFS was expected to have a positive impact on dietary intake and, consequently, support healthier behaviours and academic performance. If costs associated with the SFS and the SFP were found to be lower or generated an ICER below an acceptable threshold, this would indicate cost-effectiveness and suggest the policy was a good use of public funds. However, our findings do not support this hypothesis. While we found that the mandated schools spent less on food provision, we observed no significant differences in pupil outcomes. The interpretation of these results is, however, complicated by several methodological challenges encountered throughout the study.

The SFS is a complex public health policy embedded within a multifaceted landscape. It incurs costs across multiple sectors, affects a broad range of outcomes, and involves diverse stakeholders. These characteristics made this economic evaluation particularly challenging. While the measurement of pupil outcomes was achievable with reasonable confidence, costing was far more problematic for numerous reasons. Financial data in schools are often sensitive; schools operate within tight budgets and may be reluctant to share financial details, particularly where food provision might generate income. Furthermore, secondary school food environments are more complex than those in primary schools. Secondary school pupils have greater autonomy, and many choose to consume food purchased elsewhere. This is why one of the key limitations of the FUEL study was the inability to control for the number and nature of food outlets surrounding the schools, which may influence pupils’ dietary behaviours, making it challenging to isolate the impact of the SFS [33].

Conventional health economic methods were developed for use in clinical settings and are typically employed alongside randomised controlled trials, where data collection is highly controlled. In contrast, public health economic evaluations, such as the one presented in this paper, face considerable uncertainty and real-world constraints, and standard approaches might not always be feasible or appropriate. The FUEL study was conducted during the COVID-19 pandemic, which significantly affected collaboration with schools, recruitment, and data collection. Despite our efforts to mitigate these challenges, the already overburdened school staff were understandably limited in their capacity to engage in additional tasks, such as data collection for the FUEL study.

Due to the lack of existing evidence on the costs of food provision in schools, a micro-costing approach was deemed essential to estimate the societal cost of the SFS and the SFP. However, identifying the appropriate staff members to complete the survey was difficult, and response rates were low. In response, the costing questionnaire was shortened and converted into an online version in an attempt to boost completion rates. Despite the substantial study resources devoted to the data collection, there was still a high proportion of missing data, and the trustworthiness of the primary cost data was uncertain as many responses were incomplete. As a result, the analysis was supplemented with routine school catering expenditure data. In the absence of other evidence on the costs of school food provision, these data provided a useful complementary dataset for our analysis. It should be noted, however, that public catering expenditure data do not include all costs of food provision incurred by the schools. Therefore, some important differences across the schools might not have been captured. Furthermore, for the majority of the schools, the actual expenditure for the year 2021/2022 would be expected to be higher than in 2018/2019, even after adjusting for the CPI, due to the increase in the proportion of pupils having free school meals [49].

The primary cost data showed a wide range of reported expenditures across schools and cost categories. This variation may be attributed to reporting inaccuracies, a high proportion of missing data, but also reflect real differences in schools’ organizational and catering structures. Similar difficulties with resource-use data collection in schools have been noted in other micro-costing studies [50, 51]. With the growing number of economic evaluations of school-based interventions and a general lack of evidence on the costs of school food programs, further research is needed to identify feasible and valid approaches for collecting resource-use data in schools [34, 35, 52].

The CCA approach was selected to present a wide range of outcomes alongside disaggregated costs, which allowed for greater transparency given the diverse impacts of the SFS and the uncertainty in our data. The CCA results suggest that the SFS-mandated schools reported lower costs for items relevant to the SFS and the SFP when compared with the SFS-non-mandated schools, and this direction of difference remained the same across all analyses. However, given that the costing analysis was fraught with methodological difficulties, the magnitude of these differences should be interpreted with caution.

We also conducted an exploratory CUA. Due to the lack of baseline data, a large proportion of missingness, and uncertainty in the cost and outcome data, the results of this analysis must be interpreted cautiously. To judge the cost-effectiveness of the SFS and the SFP, an equivalent threshold for the health and social care sector of £20,000–30,000 per QALY was applied [53]. The probability of the SFS being cost-effective was high across all willingness-to-pay thresholds, ranging from 72 to 85% at the £20,000–30,000/QALY thresholds, reflecting lower intervention costs and potential health benefits, albeit with high uncertainty. However, it is important to note that this threshold reflects what society is willing to pay for a unit gain in health and the opportunity cost of a ‘health pound’. With interventions that are funded and implemented in non-health settings such as the education sector, equivalent thresholds do not exist. This is a common scenario for public health interventions and requires more research and discussion on what the appropriate threshold is for this context [54, 55].

Despite the efforts dedicated to creating a comparable sample of mandated and non-mandated schools, we found important socioeconomic differences across the two school groups that may have influenced the difference in the costs and pupil outcomes. Mandated schools had, on average, higher levels of deprivation, as indicated by higher IDACI scores. Deprivation might be associated with poorer educational performance, lower HRQoL, and poorer dietary intake independently from food provision in these schools [56–58], potentially limiting the ability of school food policies alone to shift outcomes meaningfully Also, the mandated schools had a higher proportion of pupils eligible to receive free school meals, and this might be reflected in the funding that is available to these schools to support eligible pupils.

Importantly, we found no meaningful differences in the rate of compliance with the SFS and the SFP [33]. Although exempt schools were not legally required to adhere to the SFS, they were still encouraged to do so. Over time, it has become expected that all schools are compliant, regardless of the legal status. This likely contributed to the limited observed differences in compliance and, consequently, in pupil outcomes. Despite the mandate and recent evidence highlighting low nutritional quality of food in secondary schools [59], there is currently no mechanism in place to monitor schools’ compliance with the SFS. The Department for Education and the Food Standards Agency commissioned research to support the SFS compliance pilot [60]. The pilot demonstrated feasibility of compliance checks. These will however be associated with additional costs, which will need to be considered in future analyses of the SFS, along with potential improvement in pupils’ health outcomes due to better compliance with the SFS.

### Strengths and limitations

This is the first study to evaluate the cost-effectiveness of the SFS policy in UK secondary schools. We collected a wide range of costs, including inputs from schools, catering providers, families, and volunteers, as well as nutritional, dental, HRQoL, and educational outcomes, to understand the wider impact of the SFS. The use of both primary and secondary data enhanced the depth and validity of our analysis. To address the initially low response rate to the costing survey, we adopted a flexible approach, where we revised the survey and deployed it in an online (more user-friendly) format. While our study provides important insights into the costs of food provision in secondary schools, it also highlights the broader methodological challenges of conducting economic evaluations in non-healthcare settings. Therefore, our findings need to be interpreted in light of these limitations.

First, due to the complexity of the study setting, and despite our efforts (e.g. using propensity score matching), we were unable to perfectly match the two groups of schools on all characteristics. Notably, the mandated group had a higher proportion of students from more deprived areas, which may partially explain the difference in the educational outcomes between the mandated and non-mandated schools. Second, the number of mandated schools in our sample was relatively lower, which limited our ability to draw useful comparisons across the two groups of schools. Third, primary cost and QALY data had a high proportion of missing data, which negatively impacted the reliability of our findings. Furthermore, the cost data showed a wide range of values. To mitigate this, we supplemented our analysis with secondary data on catering expenditure, and used these data for the CUA. Finally, our CUA was largely exploratory, relying on cross-sectional rather than time-series data. Since the SFS were first implemented in 2006, no data on the school costs of food provision and relevant pupil outcomes were collected, and we were not able to assess the cost-effectiveness of the SFS over time.

### Recommendations for future costing studies in school settings

Costing within health economics is often perceived as a relatively simple and straightforward process. This perception is reflected in the comparatively limited methodological literature on costing, especially when contrasted with the wealth of research on outcome measurement. In theory, costing may appear uncomplicated. However, in practice, it frequently presents substantial challenges. Our study was no exception and, in many ways, exemplifies some of the difficulties researchers can encounter.

Despite these challenges, our work generated valuable insights into how cost data collection in school settings might be approached more effectively in the future. Although we were not able to implement all of these practices within our study, these lessons can inform and support future research:

1. Collaborative cost mapping: engage early with relevant stakeholders, such as school leaders, administrators, and caterers, to jointly map out relevant cost components, ensuring a comprehensive and contextually appropriate understanding of where costs arise.
2. Understanding data systems and sources: investigate how financial data are collected and reported within schools, e.g. what budgets, accounting systems, and internal reports could be drawn upon for designing research studies.
3. Identify the right contacts: determine who within each school is best positioned to provide accurate cost data; where multiple individuals are involved, collaborative discussions should be facilitated to streamline data collection.
4. Engage and support schools: build relationships with schools to encourage participation. Consider what incentives would be meaningful for them and offer support to ease the data collection burden.
5. Invest in piloting: rigorously pilot data collection instrument with relevant stakeholders. Techniques such as think-aloud interviews can reveal practical and cognitive barriers to accurate data provision in the early stages.
6. Explore alternative sources of data and methods of data collection: in some cases, interviews may yield richer and more reliable data than surveys. Secondary data sources should be considered based on context and availability to further strengthen the validity of the findings.
7. Data verification: where possible, cross-check submitted data against other available records or conduct follow-up conversations to ensure accuracy and completeness.
8. Strengthen the role of health economics: health economics is too often considered only at the very beginning and the very end of a study, leaving little room to influence design or implementation. To improve data quality and relevance, health economists should be embedded in research teams from the outset, actively contributing to study design, instrument development, and ongoing data collection. This also calls for greater investment in a wider variety of training for health economists (e.g. in qualitative methods) and dedicated resources within grant funding to support meaningful economic evaluation.

## Conclusion

To the best of our knowledge, this is the first study that investigated the economic impact of the SFS and the SFP. The results suggest that the SFS-mandated schools spent less on food provision, while the differences in the outcomes were less pronounced. However, the difference in costs were not explained by differences in implementation of the SFS and SFP across the two school groups. Above all, this study highlighted the challenges of collecting resource-use and outcome data when conducting economic evaluations in schools. With the growing number of economic evaluations of school-based interventions, this calls for a better understanding of the appropriate methods for such evaluations.

## Supplementary Information

Supplementary Material 1.

Supplementary Material 2.

Supplementary Material 3.

Supplementary Material 4.

Supplementary Material 5.

## Data Availability

The data collected within this study is available in the manuscript and in the additional files.
