# Supplementary material for: Economic evaluation of the national school food standards across secondary schools in the Midlands, UK (the FUEL study): methodological challenges of undertaking health economics research within non-health settings
Source: Int J Behav Nutr Phys Act. 2025 Nov 12;22:142. doi: 10.1186/s12966-025-01840-6 (PMC12613869; doi:10.1186/s12966-025-01840-6)
Supplement: Supplementary file 1 — Supplementary Material 1. [file 12966_2025_1840_MOESM1_ESM.pdf]

Economic evaluation of the national school food standards across secondary schools in The Midlands, UK (the FUEL study): methodological challenges of undertaking health economics research within non-health settings

Journal: International Journal of Behavioral Nutrition and Physical Activity

Irina Pokhilenko* (0000-0001-6390-2851), Miranda Pallan (0000-0002-2868-4892), Marie Murphy (0000-00031177-1890), Peymane Adab (0000-0001-9087-3945), Breanna Morrison (0000-0002-7473-2402), Alice Sitch (0000-0001-7727-4497), Ashley Adamson (0000-0003-3735-2846), Suzanne Bartington (0000-0002-8179-7618), Rhona Duff (0000-0003-3214-524X), Tania Griffin (0000-0003-0146-4440), Kiya Hurley (0000-0002-5084-5410), Emma Lancashire (0000-0001-8601-4400), Louise McLeman (0000-0003-0329-246X), Sandra Passmore (0000-00027476-7242), Maisie Rowland (0000-0003-4762-7540), Vahid Ravaghi (0000-0003-3056-3704), Suzanne Spence (0000-0002-7089-7197), Emma Frew (0000-0002-5462-1158)

*Corresponding author, i.pokhilenko@bham.ac.uk

CHEERS 2022 Checklist

Topic	No.	Item	Location where item is reported
**Title**	1	Identify the study as an economic evaluation and specify the interventions being compared.	Cover page
**Abstract**	2	Provide a structured summary that highlights context, key methods, results, and alternative analyses.	Cover page
**Introduction**			
**Background and objectives**	3	Give the context for the study, the study question, and its practical relevance for decision making in policy or practice.	Introduction, pp 1-2
**Methods**			

**Health economic analysis plan**	4	Indicate whether a health economic analysis plan was developed and where available.	Methods, page 4
**Study population**	5	Describe characteristics of the study population (such as age range, demographics, socioeconomic, or clinical characteristics).	Methods, page 3

Topic	No.	Item	Location where item is reported
**Setting and location**	6	Provide relevant contextual information that may influence findings.	Methods, page 3
**Comparators**	7	Describe the interventions or strategies being compared and why chosen.	Introduction, page 2
**Perspective**	8	State the perspective(s) adopted by the study and why chosen.	Methods, page 4
**Time horizon**	9	State the time horizon for the study and why appropriate.	Methods, page 4
**Discount rate**	10	Report the discount rate(s) and reason chosen.	Methods, page 4
**Selection of outcomes**	11	Describe what outcomes were used as the measure(s) of benefit(s) and harm(s).	Methods, pp 7-8
**Measurement of outcomes**	12	Describe how outcomes used to capture benefit(s) and harm(s) were measured.	Methods, pp 7-8
**Valuation of outcomes**	13	Describe the population and methods used to measure and value outcomes.	Methods, pp 7-8
**Measurement and valuation of resources and costs**	14	Describe how costs were valued.	Methods, pp 4-6
**Currency, price date, and conversion**	15	Report the dates of the estimated resource quantities and unit costs, plus the currency and year of conversion.	Methods, pp 4-5

**Rationale and description of model**	16	If modelling is used, describe in detail and why used. Report if the model is publicly available and where it can be accessed.	Not applicable
**Analytics and assumptions**	17	Describe any methods for analysing or statistically transforming data, any extrapolation methods, and approaches for validating any model used.	Methods pp 5-9, Appendix
**Characterising heterogeneity**	18	Describe any methods used for estimating how the results of the study vary for subgroups.	Methods pp 8-9
**Characterising distributional effects**	19	Describe how impacts are distributed across different individuals or adjustments made to reflect priority populations.	Methods pp 8-9
**Characterising uncertainty**	20	Describe methods to characterise any sources of uncertainty in the analysis.	Methods pp 8-9
**Topic**	**No.**	**Item**	**Location where item is reported**
**Approach to engagement with patients and others affected by the study**	21	Describe any approaches to engage patients or service recipients, the general public, communities, or stakeholders (such as clinicians or payers) in the design of the study.	Study protocol
**Results**			
**Study parameters**	22	Report all analytic inputs (such as values, ranges, references) including uncertainty or distributional assumptions.	Results pp 10-20
**Summary of main results**	23	Report the mean values for the main categories of costs and outcomes of interest and summarise them in the most appropriate overall measure.	Results pp 10-20
**Effect of uncertainty**	24	Describe how uncertainty about analytic judgments, inputs, or projections affect findings. Report the effect of choice of discount rate and time horizon, if applicable.	Results, pp 19-20

**Effect of engagement with patients and others affected by the study**	25	Report on any difference patient/service recipient, general public, community, or stakeholder involvement made to the approach or findings of the study	Not applicable
**Discussion**			
**Study findings, limitations, generalisability, and current knowledge**	26	Report key findings, limitations, ethical or equity considerations not captured, and how these could affect patients, policy, or practice.	Discussion pp 20-25
**Other relevant information**			
**Source of funding**	27	Describe how the study was funded and any role of the funder in the identification, design, conduct, and reporting of the analysis	Statements and declarations
**Conflicts of interest**	28	Report authors conflicts of interest according to journal or International Committee of Medical Journal Editors requirements.	Statements and declarations

From: Husereau D, Drummond M, Augustovski F, et al. Consolidated Health Economic Evaluation Reporting Standards 2022 (CHEERS 2022) Explanation and Elaboration: A Report of the ISPOR CHEERS II Good Practices Task Force. Value Health 2022;25.
doi:10.1016/j.jval.2021.10.008
