# Supplementary material for: Economic evaluation of the national school food standards across secondary schools in the Midlands, UK (the FUEL study): methodological challenges of undertaking health economics research within non-health settings
Source: Int J Behav Nutr Phys Act. 2025 Nov 12;22:142. doi: 10.1186/s12966-025-01840-6 (PMC12613869; doi:10.1186/s12966-025-01840-6)
Supplement: Supplementary file 2 — Supplementary Material 2. [file 12966_2025_1840_MOESM2_ESM.pdf]

Economic evaluation of the national school food standards across secondary schools in The Midlands, UK (the FUEL study): methodological challenges of undertaking health economics research within non-health settings

Journal: International Journal of Behavioral Nutrition and Physical Activity

Irina Pokhilenko* (0000-0001-6390-2851), Miranda Pallan (0000-0002-2868-4892), Marie Murphy (0000-0003-1177-1890), Peymane Adab (0000-0001-9087-3945), Breanna Morrison (0000-0002-7473-2402), Alice Sitch (0000-0001-7727-4497), Ashley Adamson (0000-0003-3735-2846), Suzanne Bartington (0000-0002-8179-7618), Rhona Duff (0000-0003-3214-524X), Tania Griffin (0000-0003-0146-4440), Kiya Hurley (0000-0002-5084-5410), Emma Lancashire (0000-0001-8601-4400), Louise McLeman (0000-0003-0329-246X), Sandra Passmore (0000-0002-7476-7242), Maisie Rowland (0000-0003-4762-7540), Vahid Ravaghi (0000-0003-3056-3704), Suzanne Spence (0000-0002-7089-7197), Emma Frew (0000-0002-5462-1158)

*Corresponding author, i.pokhilenko@bham.ac.uk

Pupil survey

Pupil Survey Session 1

Information for pupil

Only the researchers will read your answers. Your name will not be used in any reports.

Part 1 Personal details

1. What is your year group?

☐ Year 7
☐ Year 9
☐ Year 10

2. What is your name?

a) First name: ____

b) Surname: ____

3. What is your date of birth? Day: __ __ Month: __ __ Year: __ __ __ __

4. What is your home postcode? (Please write in full)

____

If you have more than one address, please use the one where you spend most of your time

Part 2 About you

5. What is your sex? (Please select the option for how you identify. Tick one box only)

☐ Female
☐ Male
☐ Other (please describe) ____
☐ I would rather not say

6. **Please select <u>one group</u> from the list below that best describes you**

White

☐ English / Welsh / Scottish / Northern Irish / British
☐ Irish
☐ Gypsy or Irish Traveller
☐ Any other White background (please specify)

____

Mixed / Multiple ethnic groups

☐ White and Black Caribbean

☐ White and Black African
☐ White and Asian
☐ Any other Mixed / Multiple ethnic background (please specify)
____

Asian / Asian British

☐ Indian
☐ Pakistani
☐ Bangladeshi
☐ Chinese
☐ Any other Asian background (please specify)
____

Black / African / Caribbean / Black British

☐ African
☐ Caribbean
☐ Any other Black / African / Caribbean background (please specify)
____

Other ethnic group

☐ Arab
☐ Any other ethnic group (please specify)
____

☐ I would rather not say

7. Do you receive Free School Meals? (Tick one)

☐ Yes
☐ No
☐ I don’t know
☐ I would rather not say

Part 3 Your school canteen

8. To what extent do you agree or disagree with the following statements?

	Strongly agree	Agree	Neither agree nor disagree	Disagree	Strongly disagree	Not relevant to me
a) I feel rushed when buying food from the canteen						
b) I am given enough time to eat my lunch at school without feeling rushed						
c) There is space in the school dining area for me to sit down at a table to eat						
d) The areas where I can eat my lunch at school are clean						
e) The areas where I can eat my lunch at school are attractive						
f) The areas where I can eat my lunch at school are happy environments						
g) The areas where I can eat my lunch at school are calm environments						
h) The food and drink on offer at school looks appetising						
i) School food tastes good						
j) Teachers and staff eat in the school dining areas						

9. a) Do you ever get food or drinks for your lunch from the school canteen?

☐ Yes
☐ No [If no, please go to part 4]

9. b) On days when you get food or drinks for your lunch from the school canteen, how long do you usually queue to buy your lunch?

☐ Less than 5 mins
☐ 5-15 mins
☐ 16-30 mins
☐ Over 30 mins

9. c) To what extent do you agree or disagree with the following statements?

	Strongly agree	Agree	Neither agree nor disagree	Disagree	Strongly disagree	Not relevant to me
i. Catering staff encourage me to try new foods						
ii. Catering staff are happy						
iii. Catering staff talk to me / help me / are helpful						
iv. Catering staff encourage me to have a variety of food types (e.g. fruit/vegetables; dairy foods etc.)						
v. Catering staff put vegetables on my plate without me asking						

10. Has your school changed the types of food and drink it serves this term due to Covid-19 restrictions?

☐ Yes
☐ No
☐ I don’t know

11. If yes, please tell us how it has changed:

12. Have eating arrangements at your school changed due to Covid-19 restrictions? E.g. How long you are given for your lunch break, the spaces you are allowed to eat/drink in

☐ Yes
☐ No
☐ I don’t know

13. If yes, please tell us how they have changed:

Part 4 Does your school support healthy eating?

14. Does your school have a School Food Policy?

☐ Yes
☐ No
☐ I don’t know

15. To what extent do you agree or disagree with the following statements?

	Strongly agree	Agree	Neither agree nor disagree	Disagree	Strongly disagree
a) The school encourages healthy eating					
b) I know what to eat and drink to have a healthy diet					
c) I am confused about what to eat and drink to have a healthy diet					
d) There are signs/posters promoting healthy eating messages at school					
e) The school encourages me to drink water as my main drink					
f) I have easy access to free drinking water at school (including being able to easily fill up a water bottle brought from home)					
g) The school offers prizes for bringing in a healthy packed lunch					

16. To what extent do you agree or disagree that the school menu is easily available for you to view in advance?

☐ Strongly agree
☐ Agree
☐ Neither agree nor disagree
☐ Disagree
☐ Strongly disagree
☐ Not relevant to me

17. In the last 6 months has your school/any of your teachers used confectionery or sweets, chocolates, cakes, or biscuits as a reward?

☐ Yes
☐ No
☐ I don’t know

Part 5 Food education at your school

18. To what extent do you agree or disagree with the following statements?

	Strongly agree	Agree	Neither agree nor	Disagree	Strongly disagree
a) I learn about healthy eating and nutrition at my school					
b) I have had opportunities to learn food preparation and cooking skills during lessons in this school e.g. food technology / food science or similar					
c) I have had opportunities to learn food growing/gardening skills during lessons in this school					
d) I have had opportunities to learn food preparation and cooking skills at school clubs (outside of timetabled lesson time)					
e) I have had opportunities to learn food growing/gardening skills at school clubs (outside of timetabled lesson time)					

19. To what extent do you agree or disagree that in cooking lessons (e.g. food technology / food science or similar), you mostly learn how to cook savoury dishes?

☐ Strongly agree
☐ Agree
☐ Neither agree nor disagree
☐ Disagree
☐ Strongly disagree
☐ Not relevant to me

Part 6 School engagement with pupils

20. To what extent do you agree or disagree with the following statements?

	Strongly agree	Agree	Neither agree nor disagree	Disagree	Strongly disagree
a) There are opportunities for me to give my views on food and drink provided by school					
b) There are events, assemblies or displays about healthy eating at my school					
c) In the last 12 months, I have had the opportunity to try samples/tasters of school food					
d) There are opportunities for me to help out in the school kitchen/dining room e.g. help prepare, cook or serve the food					

Part 7 Your dental health

21. Overall, would you say that your dental health (that is the health of your teeth and mouth) is… (Tick one answer only)

☐ Very good
☐ Good
☐ Fair
☐ Poor
☐ Very poor

22. In the last three months, have you experienced any of the following?

a) Toothache ☐ Yes ☐ No

b) Sensitive tooth ☐ Yes ☐ No

c) Bleeding or swollen gums ☐ Yes ☐ No

d) A broken tooth ☐ Yes ☐ No

e) Mouth ulcers (small painful sores inside the mouth) ☐ Yes ☐ No

f) Bad breath ☐ Yes ☐ No

g) A filling ☐ Yes ☐ No

h) A decayed tooth taken out ☐ Yes ☐ No

23. Have you ever had any of the following treatments?

a)	Filling of a permanent (adult) tooth	☐ Yes	☐ No
b)	Permanent (adult) tooth taken out due to decay	☐ Yes	☐ No
c)	Filling of a milk (baby) tooth	☐ Yes	☐ No
d)	Milk (baby) tooth taken out	☐ Yes	☐ No
e)	A general anaesthetic before dental treatment (you were put to sleep during the treatment)	☐ Yes	☐ No
f)	Sedation before dental treatment (you were awake during the treatment but were given medicine to relax you before the treatment started)	☐ Yes	☐ No
g)	A brace fitted or adjusted	☐ Yes	☐ No
h)	Repair of damage to teeth after fall or other injury	☐ Yes	☐ No
i)	Scale and polish (teeth cleaned)	☐ Yes	☐ No
j)	Preventive treatment to stop teeth decaying or going bad (e.g. by painting and/or scaling the teeth)	☐ Yes	☐ No
k)	Advice on how to look after teeth (diet or tooth brushing advice)	☐ Yes	☐ No
l)	Other treatments	☐ Yes	☐ No

24. How often do you usually brush your teeth with toothpaste?

☐ Three times a day and more
☐ Twice a day
☐ Once a day
☐ Less than once a day
☐ Never

Part 8 What did you have to eat and drink yesterday?

You will now be asked to open a webpage where we will ask you about everything you had to eat and drink yesterday.

Before you complete the next survey, please answer the question(s) overleaf.

25. Were you at school yesterday?
◻ Yes – please tell us what you had to eat and drink yesterday
◻ No – please tell us about what you had to eat and drink on your most recent day at school

If yes to Q20:
25. a) Did you eat lunch yesterday?
☐ Yes
☐ No

If no to Q20:
26. a) Did you eat lunch on your most recent day at school?
☐ Yes
☐ No

26. b) (If answered yes to either questions for 21a) When did you eat your lunch?
☐ During the school lunch break
☐ During mid-morning break
☐ Any other time of the day (please specify)

Thank you for taking the time to complete this questionnaire

Follow-up

Would you like to receive a summary report of the study findings once complete?
☐ Yes
☐ No

If yes, please provide your email address (You can give your personal or school email address. This will not be stored with your answers to this questionnaire).

____

Pupil Survey Session 2

Information for pupil

Only the researchers will read your answers. Your name will not be used in any reports.

Part 1 Personal details

27. What is your year group?

☐ Year 7
☐ Year 9
☐ Year 10

28. What is your name?

a) First name: ____

b) Surname: ____

29. What is your date of birth? Day: __ __ Month: __ __ Year: __ __ __ __

30. What is your home postcode? (Please write in full)

____

If you have more than one address, please use the one where you spend most of your time

Part 2 About you

31. What is your sex? (Please select the option for how you identify. Tick one box only)

☐ Female
☐ Male
☐ Other (please write in) ____
☐ I would rather not say

32. Please select one group from the list below that best describes you

White

☐ English / Welsh / Scottish / Northern Irish / British
☐ Irish
☐ Gypsy or Irish Traveller
☐ Any other White background (please specify)

____

Mixed / Multiple ethnic groups

☐ White and Black Caribbean
☐ White and Black African
☐ White and Asian
☐ Any other Mixed / Multiple ethnic background (please specify)

____

Asian / Asian British

☐ Indian
☐ Pakistani
☐ Bangladeshi
☐ Chinese
☐ Any other Asian background (please specify)
____

Black / African / Caribbean / Black British

☐ African
☐ Caribbean
☐ Any other Black / African / Caribbean background (please specify)
____

Other ethnic group

☐ Arab
☐ Any other ethnic group (please specify): ____

☐ I would rather not say

Part 3 Travel to and from school

33. How do you usually travel to school?
☐ Walk/cycle
☐ Public transport
☐ By motor vehicle
☐ Other (please state) ____

34. Please tell us roughly how long it usually takes you to travel to school from home
☐ Less than 10 mins
☐ 10-19 mins
☐ 20-29 mins
☐ 30-59 mins
☐ 60 minutes or longer

35. How do you usually travel home from school?
☐ Walk/cycle
☐ Public transport
☐ By motor vehicle
☐ Other (please state)

36. Please tell us roughly how long it usually takes you to travel home from school
☐ Less than 10 mins
☐ 10-19 mins
☐ 20-29 mins
☐ 30-59 mins
☐ 60 minutes or longer

Part 4 Your lunch arrangements

37. In a typical school week, how often do you consume any of the following?

	Every school day	Four times a week	Three times a week	Twice a week	Once a week	Less than once a week	Never
A school dinner/lunch							
Breakfast from school canteen							
Snacks or drinks purchased from school							

If you never buy food or drinks from school, please continue to question 14.

38. Do you receive Free School Meals? (Tick one)

☐ Yes
☐ No
☐ I don't know
☐ I would rather not say

39. Roughly how much do you typically spend on food and drink purchased at school across the school day? (If you have a Free School Meal, only count spending on food and drinks purchased in addition to your Free School Meal)

☐ Less than £1
☐ £1-£2.99
☐ £3-£4.99
☐ £5-£6.99
☐ £7 or more
☐ I do not buy food and drink at school

40. In a typical school week, how often do you take any of the following with you to school?

	Every school day	Four times a week	Three times a week	Twice a week	Once a week	Less than once a week	Never
Food snacks to eat at morning or afternoon break							
Drink(s) to have at morning or afternoon break							
Packed lunch							

41. In a typical school week, how often do you do the following?

	Every school day	Four times a week	Three times a week	Twice a week	Once a week	Less than once a week	Never
Buy your lunch from local shops or takeaways to eat during the school day							
Buy a drink or a snack from a local shop or takeaway that is not part of your lunch on your way to and/or from school							

If you never buy food or drinks from local shops to have during the school day, please continue to PART 5.

42. Roughly how much do you typically spend on food and drink purchased from shops and takeaways to have during the school day?

☐ Less than £1
☐ £1-£2.99
☐ £3-£4.99
☐ £5-£6.99
☐ £7 or more
☐ I do not buy food and drink at school from local shops to have during the school day

Part 5 Questions about your general well-being and sleep

These questions ask about how you are **today.** For each question, read all the choices and decide which one is most like you **today**. Then put a tick in the box next to it like this þ. Only tick **one** box for each question.

Example

Today I feel quite upset so I will tick this box.

Upset

□ I don’t feel upset today

□ I feel a little bit upset today

□ I feel a bit upset today

☑ I feel quite upset today

□ I feel very upset today

Now think about and answer the rest of the questions below

43. Worried

☐ I don’t feel worried today
☐ I feel a little bit worried today
☐ I feel a bit worried today
☐ I feel quite worried today
☐ I feel very worried today

44. Sad

☐ I don’t feel sad today
☐ I feel a little bit sad today
☐ I feel a bit sad today
☐ I feel quite sad today
☐ I feel very sad today

45. Pain

☐ I don’t have any pain today
☐ I have a little bit of pain today
☐ I have a bit of pain today
☐ I have quite a lot of pain today
☐ I have a lot of pain today

46. Tired

☐ I don’t feel tired today
☐ I feel a little bit tired today
☐ I feel a bit tired today
☐ I feel quite tired today
☐ I feel very tired today

47. Annoyed

☐ I don’t feel annoyed today
☐ I feel a little bit annoyed today
☐ I feel a bit annoyed today
☐ I feel quite annoyed today
☐ I feel very annoyed today

48. School Work/Homework (such as reading, writing, doing lessons)

☐ I have no problems with my schoolwork/homework today
☐ I have a few problems with my schoolwork/homework today
☐ I have some problems with my schoolwork/homework today
☐ I have many problems with my schoolwork/homework today
☐ I can’t do my schoolwork/homework today

49. Sleep

☐ Last night I had no problems sleeping
☐ Last night I had a few problems sleeping
☐ Last night I had some problems sleeping
☐ Last night I had many problems sleeping
☐ Last night I couldn’t sleep at all

50. Daily routine (things like eating, having a bath/shower, getting dressed)

☐ I have no problems with my daily routine today
☐ I have a few problems with my daily routine today
☐ I have some problems with my daily routine today
☐ I have many problems with my daily routine today
☐ I can’t do my daily routine today

51. Able to join in activities (things like playing out with your friends, doing sports, joining in things)

☐ I can join in with any activities today
☐ I can join in with most activities today
☐ I can join in with some activities today
☐ I can join in with a few activities today
☐ I can join in with no activities today

52. Roughly, what time did you fall asleep last night? (We are interested in the time you went to sleep, not the time you went to bed) _______________________________

53. Roughly, what time did you wake up this morning? (We are interested in the time you woke up, not when you got out of bed) _______________________________

Part 6 What did you have to eat and drink yesterday?

You will now be taken to another webpage where we will ask you about everything you had to eat and drink yesterday.

Before you complete the next survey, please answer the question(s) below.

54. Were you at school yesterday?

☐ Yes – please tell us what you had to eat and drink yesterday
☐ No – please tell us about what you had to eat and drink on your most recent day at school

If yes to Q28:

55. Did you eat lunch yesterday?

☐ Yes
☐ No

If no to Q28:

29. Did you eat lunch on your most recent day at school?

☐ Yes

☐ No

30. (If yes to either versions of question 29) When did you eat your lunch?

☐ During the school lunch break

☐ During mid-morning break

☐ Any other time of the day (please specify)

Thank you for taking the time to complete this questionnaire

Follow-up

Would you like to receive a summary report of the study findings once complete?

☐ Yes

☐ No

If yes, please provide your email address (You can give your personal or school email address. This will not be stored with your answers to this questionnaire).

____
