# Supplementary material for: Economic evaluation of the national school food standards across secondary schools in the Midlands, UK (the FUEL study): methodological challenges of undertaking health economics research within non-health settings
Source: Int J Behav Nutr Phys Act. 2025 Nov 12;22:142. doi: 10.1186/s12966-025-01840-6 (PMC12613869; doi:10.1186/s12966-025-01840-6)
Supplement: Supplementary file 4 — Supplementary Material 4. [file 12966_2025_1840_MOESM4_ESM.pdf]

Economic evaluation of the national school food standards across secondary schools in The Midlands, UK (the FUEL study): methodological challenges of undertaking health economics research within non-health settings

Journal: International Journal of Behavioral Nutrition and Physical Activity

Irina Pokhilenko* (0000-0001-6390-2851), Miranda Pallan (0000-0002-2868-4892), Marie Murphy (00000003-1177-1890), Peymane Adab (0000-0001-9087-3945), Breanna Morrison (0000-0002-7473-2402), Alice Sitch (0000-0001-7727-4497), Ashley Adamson (0000-0003-3735-2846), Suzanne Bartington (0000-00028179-7618), Rhona Duff (0000-0003-3214-524X), Tania Griffin (0000-0003-0146-4440), Kiya Hurley (0000-0002-5084-5410), Emma Lancashire (0000-0001-8601-4400), Louise McLeman (0000-0003-0329-246X), Sandra Passmore (0000-0002-7476-7242), Maisie Rowland (0000-0003-4762-7540), Vahid Ravaghi (0000-0003-3056-3704), Suzanne Spence (0000-0002-7089-7197), Emma Frew (0000-0002-5462-1158)

*Corresponding author, i.pokhilenko@bham.ac.uk

School Food: Costings Survey (first, longer version)

Information for respondents

We hope to gather information on the costs associated with:

- The school eating and drinking facilities
- Providing school food to pupils and staff
- Teaching and learning about food, cooking, growing, health and healthy eating
- School food governance

This will help us to estimate the cost of school food provision and food education.
Please complete the following questions to the best of your ability, seeking the advice of other school or catering staff e.g. business manager, catering manager as required.
Please return to the FUEL study team in person or by scanned/emailed copy to fuelstudy@contacts.bham.ac.uk.

The questions will start on the next page.
List of sections

**General questions (all to answer)**
• Places to purchase and consume food and drink
• Catering providers
**Dining environment**
• Your cashless payment system
**Food Provision**
• Your water fountains
• School clubs in which food is served
• Offering tasting opportunities / samples to pupils, parents or staff
• Food rewards for good behaviour
• Prizes and other incentives for bringing in a healthy packed lunch
**Food Education**
• Growing food
•
• School gardening clubs
• Cookery classes in the curriculum
• School cooking clubs
• A qualified subject specialist to teach food education
• Healthy eating events
**School Food Governance**
• Your school Healthy Eating Award
• Your School Food Policy
• Subsidising school food
• Pupil Premium spend on healthy eating
• Independent monitoring of school food
• Training for school staff on health and well-being

GENERAL QUESTIONS

Places to purchase and consume food and drink

1. Please tell us more about all the food outlets within your school, both indoor and outdoor (include canteens, kiosks, coffee shops and tuck shops in the school, outdoor areas on site and sixth form, if applicable). If you need more space, please use the box on the last page of the survey

Name of outlet	No. staff* at lunch	No. staff* at break	No. staff* at breakfast	Number of food service points**	Number of till points	Seating capacity (if applicable)	Is this outlet cashless? (y/n)	Is the school financially responsible for maintaining the outlet?(y/n)
1.								
2.								
3.								
4.								
5.								
6.								

* Include staff involved in serving or supervising

**Food service points are those in which hot or cold food is served – this includes by a catering staff member or self-service

2. Please tell us more about the dining areas/rooms in your school. A dining area is defined as an area with table and chairs for eating, including areas for packed lunches and outdoor areas on site. (If you need more space, please use the box on the last page of the survey)

Name of dining area	No. staff supervising at lunch	No. staff supervising at break	No. staff supervising at breakfast	Seating capacity	Is the school financially responsible for maintaining the area? (y/n)
1.					
2.					
3.					
4.					
5.					
6.					

If you are unable to complete this section, please ask the appropriate member of staff to complete it. Alternatively, you can provide us with the name/job title (and email address if you have it) of the appropriate staff member, and we will we contact them to complete it:

3. Are pupils allowed to eat in classrooms at your school?

☐ Yes
☐ No

4. Please tell us about the vending machines your school has, if any

	Number
How many are there in total?	
How many are cashless?	
How many is the school financially responsible for maintaining?	

5. Have you made any alterations to the food outlets or dining area(s) to make them more appealing to pupils over the last five years? (Please cross)

☐ Yes
☐ No

6. If yes, what alterations have you made and at what cost? (If you need more space, please use the box on the last page of the survey)

Description of change	Estimate of cost	Date of change
	£	
	£	
	£	
	£	
	£	

7. Who pays for the physical maintenance costs of the food outlets and dining areas? (Please cross all that apply)

☐ The school
☐ The catering providers
☐ Both
☐ Other (please specify):

8. What proportion of maintenance costs for food outlets and dining areas is covered by the school? (please provide a percentage)

__________%

If you are unable to complete this section, please ask the appropriate member of staff to complete it. Alternatively, you can provide us with the name/job title (and email address if you have it) of the appropriate staff member, and we will we contact them to complete it:

Catering providers

9. What is the total annual cost of the catering contract?

   £

10. Please tell us about what catering roles there are at the school and how many staff perform these roles (if you need more space, please use the box on the last page of the survey)

Description of role	No. staff in role	Total hours per week across all staff in this role
1.		
2.		
3.		
4.		

If you are unable to complete this section, please ask the appropriate member of staff to complete it. Alternatively, you can provide us with the name/job title (and email address if you have it) of the appropriate staff member, and we will we contact them to complete it:

DINING ENVIRONMENT
Your cashless payment system

Only complete this section if your school uses a cashless payment system for food and drink purchasing

11. What type of cashless system does your school use for food and drink purchasing? (Please cross all that apply)

☐ Finger print technology
☐ Loaded card with PIN
☐ Loaded card with contactless
☐ Other (please specify):

12. What is the name of the provider of your cashless payment system?

13. Does your school have to pay an ongoing (maintenance) cost to have a cashless payment system?

☐ Yes

☐ No

14. If yes, please state the annual maintenance costs (You can provide a rough estimate if you do not have a precise figure)

£

20.

If you are unable to complete this section, please ask the appropriate member of staff to complete it. Alternatively, you can provide us with the name/job title (and email address if you have it) of the appropriate staff member, and we will we contact them to complete it:

FOOD PROVISION
Your water fountains

Only complete this section if your school has water fountains.

15. How many water fountains does the school have?

16. How many water fountains are currently operational/functioning?

17. Who is responsible for covering the cost of maintenance of water fountains? (Please cross all that apply)

☐ The school
☐ The catering provider
☐ Other (please specify):

If you are unable to complete this section, please ask the appropriate member of staff to complete it. Alternatively, you can provide us with the name/job title (and email address if you have it) of the appropriate staff member, and we will we contact them to complete it:

School clubs in which food is served

Only complete this section if you school runs clubs in which food or drink is served

Breakfast clubs during which food or drink is served to pupils

18. Does your school have a breakfast club?

☐ Yes
☐ No [go straight to question 31]

19. Please estimate the average total <u>weekly</u> spend on food and drink for school breakfast clubs at which food and drink is served, **excluding cookery clubs**. (You can provide a rough estimate if you do not have a precise figure)

    £

20. Who covers the cost of food and drink served at breakfast clubs? (Please cross all that apply)

☐ The school
☐ The parents
☐ The catering providers
☐ Other (please specify):

After school clubs during which food or drink is served to pupils

21. Does your school have an after school club during which food or drink is served?

☐ Yes
☐ No [go straight to the next section on page 12]

22. Please estimate the average total <u>weekly</u> spend on food and drink for after school clubs at which food and drink is served, **excluding cookery clubs**. (You can provide a rough estimate if you do not have a precise figure)

    £

23. Who covers the cost of food and drink served at after school clubs? (Please cross all that apply)

☐ The school
☐ The parents
☐ The catering providers
☐ Other (please specify):

If you are unable to complete this section, please ask the appropriate member of staff to complete it. Alternatively, you can provide us with the name/job title (and email address if you have it) of the appropriate staff member, and we will we contact them to complete it:

Offering tasting opportunities / samples to pupils, parents or staff

Only complete this section if your school offers tasting opportunities / samples to pupils, parents or staff. This includes informal tasting opportunities in food outlets or larger-scale taster days/events.

24. How many tasting opportunities do you offer to pupils, parents or staff in a typical school year?

    Number:

25. Please estimate any school-related costs (if any) from running each of these events (You can provide a rough estimate if you do not have a precise figure):

26. Who covers the cost of tasting opportunities / samples to pupils, parents or staff? (Please cross all that apply)

☐ The school
☐ The catering providers
☐ Other (please specify):

If you are unable to complete this section, please ask the appropriate member of staff to complete it. Alternatively, you can provide us with the name/job title (and email address if you have it) of the appropriate staff member, and we will we contact them to complete it:

Food rewards for good behaviour

Only complete this section if your school provides food rewards for good behaviour. This section relates only to offering high sugar or high fat foods/treats as rewards to pupils e.g. sweets, chocolate, pizza.

27. What is the <u>annual</u> spend on high sugar foods/treats for rewards for pupils? (You can provide a rough estimate if you do not have a precise figure)

    £

28. Who covers the cost of rewards? (Please cross all that apply)

☐ The school
☐ The teacher
☐ The catering providers
☐ Other (please specify):

If you are unable to complete this section, please ask the appropriate member of staff to complete it. Alternatively, you can provide us with the name/job title (and email address if you have it) of the appropriate staff member, and we will we contact them to complete it:

Prizes and other incentives for bringing in a healthy packed lunch

Only complete this section if your school provides prizes and other incentives for bringing in a healthy packed lunch.

29. What is the annual spend on prizes and other incentives for bringing in a healthy packed lunch?. (You can provide a rough estimate if you do not have a precise figure)

£

30. Who covers the cost of rewards/prizes? (Please cross all that apply)

☐ The school
☐ The teacher
☐ The catering providers
☐ Other (please specify):

If you are unable to complete this section, please ask the appropriate member of staff to complete it. Alternatively, you can provide us with the name/job title (and email address if you have it) of the appropriate staff member, and we will we contact them to complete it:

FOOD EDUCATION
Growing food

Only complete this section if your school has a gardening area for growing food.

31. What is the <u>annual</u> cost of maintaining the food growing area(s)? (You can provide a rough estimate if you do not have a precise figure)

    £

32. Does the catering provider use any of the food grown on-site in its school meals provision?

☐ Yes
☐ No
☐ I don't know

33. Who is responsible for covering the cost of maintaining the food growing area(s)? (Please cross all that apply)

☐ The school
☐ The catering provider
☐ Other (please specify):

34. Do you use staff or community / parent volunteers to support any food growing activities?

☐ Yes
☐ No

35. Please estimate the average <u>weekly</u> hours each group typically spends supporting food growing activities

	Weekly staff hours	Weekly volunteer hours
During food-growing season (Spring through to Autumn)		
Out of food-growing season (Winter months)		

36. For staff, is this time spent in addition to, or as part of, their contracted hours?

☐ In addition to contracted hours
☐ Part of contracted hours
☐ Not applicable

If you are unable to complete this section, please ask the appropriate member of staff to complete it. Alternatively, you can provide us with the name/job title (and email address if you have it) of the appropriate staff member, and we will we contact them to complete it:

School gardening clubs

Only complete this section if your school offers gardening clubs to pupils and/or parents. This section refers to extracurricular gardening clubs only.

37. Please tell us about the gardening club sessions the school delivers for each group. This section refers to extracurricular gardening clubs only.

	How many sessions* per year?	Typical number of attendees each session
Year 7		
Year 8		
Year 9		
Year 10		
Year 11		
Post-16		
Parents only		

* When counting, include any sessions that are repeated (i.e. if the programme runs multiple times in the year)

38. Who delivers your gardening clubs to pupils and/or parents? (Please cross all that apply)

☐ School staff
☐ Volunteers
☐ External organisation/agency
☐ Other (please specify):

39. Do pupils/parents pay a fee to attend? (Please cross)

☐ Yes
☐ No

40. If pupils/parents pay a fee to attend, how much do they pay per session?

£

41. If you use an **external agency** to deliver these clubs, how is this paid for? (please cross all that apply)

☐ School pays some/whole costs
☐ Pupils/ parents pay some/whole costs
☐ Not applicable

42. If the school pays some/all costs to an external agency, what is the average cost of this service to the school per session or per year? (You can provide a rough estimate if you do not have a precise figure)

£ per session/year* (*please delete as appropriate)

43. If you deliver the clubs **in-house** (i.e. using your own staff), what is the additional cost per session delivered (e.g. for extra payments to staff, plants, equipment)?

£

44. Does the school subsidise/waive the attendance fees for particular groups of pupils e.g. those with Pupil Premium funding? (Please cross)

☐ Yes
☐ No

45. If yes, please provide more detail on what groups of pupils are eligible to receive support for attendance fees:

46. Please estimate the number of pupils receiving support for attendance fees (total across the whole school per year)

Number:

If you are unable to complete this section, please ask the appropriate member of staff to complete it. Alternatively, you can provide us with the name/job title (and email address if you have it) of the appropriate staff member, and we will we contact them to complete it:

Cookery classes in the curriculum

Only answer this section if your school delivers practical food preparation/cookery lessons in the curriculum. This section refers to food preparation / cooking lessons delivered as part of the curriculum only. These may be referred to as Food Science, Food Technology, Cooking and Nutrition, Nutrition Science, Home Economics, or something similar.

47. Please tell us about the practical **food preparation/cooking lessons** delivered at your school as part of the school curriculum for each year group e.g. Food Science, Food Technology, Nutrition Science, Cooking and Nutrition, Home Economics, etc.? You should also state who delivers these lessons e.g. a teacher, another member of staff or an external organisation.

	No. lessons per pupil (over a year)	No. pupils receiving lessons (over a year)	Who delivers these lessons?	Name of subject	Curriculum / syllabus / exam board followed
Year 7					

Year 8					
Year 9					
Year 10					
Year 11					
Post-16					

48. If you use an **external agency** to deliver these lessons, what is the average annual cost of this service? (You can provide a rough estimate if you do not have a precise figure)

    £

49. If you deliver lessons **in-house** (i.e. using your own staff), what is the additional cost from delivering each food preparation/cookery lesson, in terms of:

Staff costs/time? £
Equipment? £
Subsidising/waiving attendance fees? £
Other (please specify): £
(You can provide a rough estimate if you do not have a precise figure)

50. Do pupils cover the cost of their own ingredients? (Please cross)

☐ Yes
☐ No

51. How much do ingredients typically cost per lesson per pupil? (Please provide a range if you would prefer)

    £

52. Does the school subsidise the purchasing of ingredients for particular groups of pupils e.g. those with Pupil Premium funding? (Please cross)

☐ Yes
☐ No

53. If yes, please provide more detail on what groups of pupils are eligible to receive support to purchase ingredients:

54. Please estimate the number of pupils receiving support to purchase ingredients (total across the whole school per year)

    Number:

If you are unable to complete this section, please ask the appropriate member of staff to complete it. Alternatively, you can provide us with the name/job title (and email address if you have it) of the appropriate staff member, and we will we contact them to complete it:

School cooking clubs

Only answer this section if the school offers cooking clubs for pupils and/or parents. This section refers to extracurricular cooking clubs only.

55. Please tell us about any cooking club sessions the school delivers for each group. This section refers to extracurricular food preparation/cooking clubs only.

	How many sessions per year?	Typical number of attendees each session
Year 7		
Year 8		
Year 9		
Year 10		
Year 11		
Post-16		
Parents only		

* When counting, include any sessions that are repeated (i.e. if the programme runs multiple times in the year)

56. Who delivers your cooking clubs to pupils and/or parents? (Please cross)

☐ School staff
☐ Volunteers
☐ External organisation/agency
☐ Other (please specify):

57. Do pupils/parents pay a fee to attend? (Please cross)

☐ Yes
☐ No

58. If pupils/parents pay a fee to attend, how much do they pay per session?

£

59. If you use an **external agency** to deliver these clubs, how is this paid for? (please cross all that apply)

☐ School pays some/whole costs
☐ Pupils/ parents pay some/whole costs
☐ Not applicable

60. If the school pays some/all costs, what is the average cost of this service to the school per session/programme? (You can provide a rough estimate if you do not have a precise figure)

£ per session/programme* (*please delete as appropriate)

61. If you deliver the clubs **in-house** (i.e. using your own staff), what is the cost from delivering each session in terms of:

Extra payments for staff £
Equipment? £
Subsidising/waiving attendance fees? £
Other (please specify): £

62. Does the school subsidise the attendance fees/purchasing of ingredients for particular groups of pupils e.g. those with pupil premium funding? (Please cross)

☐ Yes
☐ No

63. If yes, please provide more detail on what groups of pupils are eligible to receive support for attendance fees/purchasing of ingredients:

64. Please estimate the number of pupils receiving support for attendance fees/purchasing of ingredients (total across the whole school per year)

Number:

If you are unable to complete this section, please ask the appropriate member of staff to complete it. Alternatively, you can provide us with the name/job title (and email address if you have it) of the appropriate staff member, and we will we contact them to complete it:

A qualified subject specialist to teach food education

Only answer this section if your school has a qualified subject specialist to teach food education.

65. How many members of staff have a qualification for teaching food education e.g. Food Technology PGCE; Design and Technology PGCE?

    Number:

If you are unable to complete this section, please ask the appropriate member of staff to complete it. Alternatively, you can provide us with the name/job title (and email address if you have it) of the appropriate staff member, and we will we contact them to complete it:

Healthy eating events

Only answer this section if your school delivers healthy eating events in a typical school year. This could be a one-off session or a range of sessions delivered over an extended period, such as a week or month. This could include nationally-organised events e.g. BNF Healthy Eating Week; LACA National School Meals Week; or those organised more locally or by the school alone.

66. Please provide some further details on the types of events your school holds and costs associated e.g. staff time, equipment hire, sessions, marketing materials, etc. (You can provide a rough estimate if you do not have a precise figure)

Brief description of session	No. sessions	Total cost	Who covers cost?
		£	
		£	
		£	
		£	
		£	
		£	
		£	
		£	
		£	
		£	
		£	

If you are unable to complete this section, please ask the appropriate member of staff to complete it. Alternatively, you can provide us with the name/job title (and email address if you have it) of the appropriate staff member, and we will we contact them to complete it:

Other activities to promote healthy eating (all to answer)

67. Please tell us about anything else your school does to promote or support healthy eating, cooking or food growing:

If you are unable to complete this section, please ask the appropriate member of staff to complete it. Alternatively, you can provide us with the name/job title (and email address if you have it) of the appropriate staff member, and we will we contact them to complete it:

SCHOOL FOOD GOVERNANCE
Your school Healthy Eating Award

Only answer this section if your school has a Healthy Eating Award.

68. Which of the following awards have been achieved by the school? (Please cross all that apply)

☐ Soil Association Food for Life Schools Award – Gold
☐ Soil Association Food for Life Schools Award – Silver
☐ Soil Association Food for Life Schools Award – Bronze
☐ Soil Association Food for Life Schools Award – Served Here / Catering Mark
☐ Healthy Schools Rating Scheme – Gold
☐ Healthy Schools Rating Scheme - Silver
☐ Healthy Schools Rating Scheme - Bronze
☐ LACA Award for Excellence
☐ Other (please specify):

69. Did your school need to pay a fee to receive the award(s)? (Please cross)

☐ Yes, a one off fee
☐ Yes, a regular fee to retain the award
☐ No

70. If yes, what was/is the fee? (You can provide a rough estimate if you do not have a precise figure. If you have multiple awards, please provide the figure for each one separately)

£

71. What <u>additional</u> costs to the school, if any, were associated with achieving this award? (You can provide a rough estimate if you do not have a precise figure)

72. Who covered the costs of achieving the award? (Please tick all that apply)

☐ The school
☐ The catering provider
☐ Other (please specify):

If you are unable to complete this section, please ask the appropriate member of staff to complete it. Alternatively, you can provide us with the name/job title (and email address if you have it) of the appropriate staff member, and we will we contact them to complete it:

Your School Food Policy

Only answer this section if your school has a written School Food Policy.

73. Please estimate how much staff time is spent developing or reviewing the school food policy each year. (You can provide a rough estimation if you do not have a precise figure)

    Amount of time:

If you are unable to complete this section, please ask the appropriate member of staff to complete it. Alternatively, you can provide us with the name/job title (and email address if you have it) of the appropriate staff member, and we will we contact them to complete it:

Subsidising school food

Only complete this section if you offer subsidies for some pupils or staff groups to have a school lunch.

74. Please tell us about the subsidies / discounts you offer to any of the following pupil groups:

Group	Subsidies offered (y/n)	What discount?	How many pupils?
Year 7 pupils			
Families with more than one child at school			
Children purchasing school lunch every day			
Staff			
Other group (please describe):			

75. Please tell us about any other subsidies / funding you offer to support pupils or staff to access food at school (including breakfast or an evening meal):

If you are unable to complete this section, please ask the appropriate member of staff to complete it. Alternatively, you can provide us with the name/job title (and email address if you have it) of the appropriate staff member, and we will we contact them to complete it:

Pupil Premium spend on healthy eating

Only answer this section if your school uses Pupil Premium funds on healthy eating activities.

76. Does your school use Pupil Premium funds for any of the following activities?

☐ Breakfast clubs [please answer question 98]
☐ After school clubs [please answer question 99]
☐ Other activities relating to healthy eating [please answer question 100]

77. How much of the Pupil Premium budget is spent on breakfast clubs?

78. How much of the Pupil Premium budget is spent on after school clubs?

79. How much of the Pupil Premium budget is spent on other activities relating to healthy eating? (Please specify nature of activity)

If you are unable to complete this section, please ask the appropriate member of staff to complete it. Alternatively, you can provide us with the name/job title (and email address if you have it) of the appropriate staff member, and we will we contact them to complete it:

Other funding to support healthy eating (all to answer)

80. Please tell us about any other funding sources that support school food provision or food education (including funding source, value of funding and how this is spent):

If you are unable to complete this section, please ask the appropriate member of staff to complete it. Alternatively, you can provide us with the name/job title (and email address if you have it) of the appropriate staff member, and we will we contact them to complete it:

Independent monitoring of school food

Only answer this section if you have used an independent service to monitor school food.

81. Have you sought expert and/or independent support for any of the following tasks? (You can provide a rough estimate if you do not have a precise figure)

	Y / N	Cost to school
Drafting the catering contract		£
Monitoring compliance with the School Food Standards		£
Monitoring compliance with the Government Buying Standards		£
Improving school food quality and take-up		£
Teaching pupils about healthy eating or food preparation e.g. chefs		£
Other (please specify):		£

If you are unable to complete this section, please ask the appropriate member of staff to complete it. Alternatively, you can provide us with the name/job title (and email address if you have it) of the appropriate staff member, and we will we contact them to complete it:

Training for school staff on health and well-being

Only answer this section if your school staff undertake training on health and well-being in a typical school year. This section refers only to training on health and well-being specifically. This applies to teaching staff and any other school staff, including catering staff that are directly employed by the school (but not catering staff who are employed by external catering contractors). This may include internal or external workshops, qualifications/certificates, online training, webinars and attendance at conferences. Some examples may include the British Nutrition Foundation webinars, LACA seminars etc.

82. What type of health and well-being training is undertaken by staff in a typical school year? (You can provide a rough estimate of cost if you do not have a precise figure)

Description of training	No. sessions	Total cost	Classroom cover required? (y/n)	Number of staff attending	Who covers the cost?
Workshop –external provider		£			
Workshop –in-house		£			
Course –external provider		£			
Course –in-house		£			
Formal qualification / certification		£			
Online training course		£			
Webinar		£			
Conference		£			
Other (please specify):		£			

Thank you for taking the time to complete the questionnaire

If there is anything additional you would like to tell us in relation to school food, please do so by writing in the space below. If you have nothing further to add please leave the space blank.

School costing survey (new online version)

GENERAL QUESTIONS Catering providers

1. Does your school have external caterers?

☐ Yes (please go to question 2)

☐ No (please go to question 3)

2. What is the total <u>annual cost</u> of the catering contract?

£

Places to purchase and consume food and drink

3. How many food outlets and dining areas are there within your school? Please think about both indoor and outdoor food outlets and dining areas (include canteens, kiosks, coffee shops and tuck shops in the school, outdoor areas on site and sixth form, if applicable).

4. Who is financially responsible for maintaining the food outlets and dining areas within your school? Please select one option.

   ☐ The school (please go to question 6)

   ☐ The catering provider (please go to question 6)

   ☐ Both the school and the catering provider (please go to question 5) ☐
   Other (please specify and go to question 6):

5. What proportion of costs associated with maintaining the food outlets and dining areas is covered by the school?

%

6. Please tell us about the staff involved in food preparation, serving, and meal supervision on a typical school day.

Type of staff	School employed or caterer employed?	Number of staff needed per day	Total hours spent on food preparation, serving or meal supervision per day (for each staff group)
Example: Catering staff	*School employed*	*5*	*10 hours*
Catering staff			
Teaching staff			
Other staff (please specify):			

7. Does your school have vending machines?

☐ Yes (please go to question 8)
☐ No (please go to question 10)

8. How many vending machines are there in your school?

9. Is the school financially responsible for maintaining vending machines?

☐ Yes, all vending machines (please go to question 11)
☐ Yes, some vending machines (please go to question 10)
☐ No (please go to question 11)

10. How many vending machines is the school financially responsible for?

Alterations to food outlets and dining areas

We are interested in any substantial changes (and associated costs) that your school has made related to increasing uptake of school meals and/or improving the lunchtime experience for pupils.

11. Have you made any alterations to the food outlets or dining area(s) related to improving the lunchtime experience for pupils/increasing uptake of school meals over the last five years?

☐ Yes (please go to question 12)
☐ No (please go to question 13)

12. Please describe these changes and provide an estimate of the costs associated with the changes including one-off (e.g. set-up) costs and annual ongoing costs.

Description of change	Estimate of any one-off costs	Estimate of any on-going costs
	£	£
	£	£
	£	£
	£	£

13. Can you think of any other additional ongoing costs for improving the lunchtime experience for pupils/increasing uptake of school meals over and above what has already been listed? Please describe these costs and estimate an annual amount.

Other ongoing costs	Annual estimate of cost
	£
	£
	£
	£

If you are unable to complete this section, please ask the appropriate member of staff to complete it. Alternatively, you can provide us with the name/job title (and email address if you have it) of the appropriate staff member, and we will we contact them to complete it:

FOOD EDUCATION Growing food

14. Does your school have an area for growing food?

☐ Yes (please go to question 15)

☐ No (please go to question 20)

15. What is the <u>annual cost</u> of maintaining the food growing area(s)? You can provide a rough estimate if you do not have a precise figure.

£

16. Who is responsible for covering the cost of maintaining the food growing area(s)? Please select one option.

☐ The school (please go to question 18)

☐ The catering provider (please go to question 18)

☐ Both the school and the catering provider (please go to question 17)

☐ Other (please specify and go to question 18):

17. What proportion of costs associated with maintaining the food growing areas is covered by the school?

%

18. Does the catering provider use any of the food grown on-site in its school meals provision?

☐ Yes

☐ No

☐ I don't know

19. Please tell us about the staff and/or volunteers involved in food growing activities at the school including delivery of activities and garden maintenance in a typical week.

Type of staff	Number of staff/volunteers needed per week	Total hours spent per week on food growing activities/garden maintenance (for each staff/volunteer group)
School-employed staff		

Volunteers		
External agency staff		
Other staff (please specify):		

School gardening clubs

20. Does your school offer extracurricular gardening clubs to pupils/parents?

☐ Yes (please go to question 21)

☐ No (please go to question 22)

21. Please explain and list the costs associated with the club e.g. costs to parents, pupils, the school, external agencies, etc.

Cookery classes in the curriculum

22. Does your school deliver practical food preparation/cookery lessons in the curriculum? These may be referred to as Food Science, Food Technology, Cooking and Nutrition, Nutrition Science, Home Economics, or something similar.

☐ Yes (please go to question 23)

☐ No (please go to question 28)

23. Please tell us about the staff involved in delivering practical food preparation/cookery lessons.

Type of staff	Number of staff employed	Total hours spent per week delivering the food curriculum (for each staff group)
Qualified food education staff (e.g. Food Technology PGCE; Design and Technology PGCE?		

Other staff (please specify):		

24. If you use an external agency to deliver these lessons, what is the average annual cost of this service? You can provide a rough estimate if you do not have a precise figure.

£

25. Do pupils/parents cover the cost of their own ingredients?

☐ Yes
☐ No

26. Does the school subsidise the purchasing of ingredients for particular groups of pupils e.g. those with Pupil Premium funding?

☐ Yes
☐ No

27. If yes, please provide more detail on what groups of pupils are eligible to receive support to purchase ingredients.

School cooking clubs

28. Does your school offer extracurricular food preparation/cooking clubs for pupils and/or parents?

☐ Yes (please go to question 29)
☐ No (please go to question 39)

29. Please provide a brief description of extracurricular food preparation/cooking clubs offered at your school.

30. Please describe the staff/volunteers involved in the delivery of food preparation/cooking clubs at your school.

Type of staff	Number of staff needed per week	Total hours spent on food preparation/cooking clubs (for each staff/volunteer group)
School-employed staff		
Volunteers		
External agency staff		
Other staff (please specify):		

31. Do pupils/parents pay a fee to attend?

☐ Yes

☐ No

32. If pupils/parents pay a fee to attend, how much do they pay per session?

£

33. If you use an external agency to deliver these clubs, how is this paid for? Please select all that apply.

☐ School pays some/whole costs

☐ Pupils/ parents pay some/whole costs

☐ Not applicable

34. If the school pays some/all costs, what is the <u>average cost</u> of this service to the school per session/programme? You can provide a rough estimate if you do not have a precise figure.

£	☐ Per session<br>☐ Per programme

35. If you deliver the clubs in-house (i.e. using your own staff), what is the cost of delivering each session in terms of:

Equipment	£
Subsidising/waiving attendance fees?	£
Other (please specify):	£

36. Does the school subsidise the attendance fees/purchasing of ingredients for particular groups of pupils e.g. those with pupil premium funding?

☐ Yes (please go to question 37)

☐ No (please go to question 39)

37. If yes, please provide more detail on what groups of pupils are eligible to receive support for attendance fees/purchasing of ingredients.

38. Please estimate the number of pupils receiving support for attendance fees/purchasing of ingredients (total across the whole school per year).

Healthy eating events

39. Does the school run healthy eating events? This could be a one-off session or a range of sessions delivered over an extended period, such as a week or month. This could include nationallyorganised events e.g. BNF Healthy Eating Week; LACA National School Meals Week; or those organised more locally or by the school alone.

☐ Yes (please go to question 40)

☐ No (please go to question 41)

40. Please provide some further details on the types of events your school holds, and annual costs associated with e.g. staff time, equipment hire, sessions, marketing materials, etc. You can provide a rough estimate if you do not have a precise figure.

Brief description of session	Total annual cost	Who covers the cost?
	£	
	£	
	£	
	£	
	£	
	£	

Other activities to promote healthy eating

41. Please tell us about anything else your school does to promote or support healthy eating, cooking or food growing and provide an annual estimate of costs spent on these activities.

Brief description of activity	Total annual cost	Who covers cost?
	£	
	£	
	£	
	£	
	£	
	£	

	£	

SCHOOL FOOD GOVERNANCE Your school Healthy Eating Award

42. Does your school have a Healthy Eating Award?

☐ Yes (please go to question 43)

☐ No (please go to question 49)

43. Which of the following awards have been achieved by the school? Please select all that apply.

☐ Soil Association Food for Life Schools Award – Gold

☐ Soil Association Food for Life Schools Award – Silver

☐ Soil Association Food for Life Schools Award – Bronze

☐ Soil Association Food for Life Schools Award – Served Here / Catering Mark

☐ Healthy Schools Rating Scheme – Gold

☐ Healthy Schools Rating Scheme - Silver

☐ Healthy Schools Rating Scheme - Bronze

☐ LACA Award for Excellence ☐ Other
(please specify):

44. Did your school need to pay a fee to receive the award(s)?

☐ Yes, a one-off fee

☐ Yes, a regular fee to retain the award

☐ No

45. If yes, what was/is the fee? You can provide a rough estimate if you do not have a precise figure. If you have multiple awards, please provide the figure for each one separately.

46. What additional costs to the school, if any, were associated with achieving this award beyond what has already been captured? You can provide a rough estimate if you do not have a precise figure.

47. Who covered the costs of achieving the award? Please select one option.

☐ The school (please go to question 49)

☐ The catering provider (please go to question 49)

☐ Both the school and the catering provider (please go to question 48) ☐
Other (please specify and go to question 49):

48. What proportion of costs associated with achieving the award was covered by the school?

%

Your School Food Policy

49. Does your school have a written School Food Policy?

☐ Yes (please go to question 50)

☐ No (please go to question 51)

50. If yes, please estimate how much staff time is spent developing or reviewing the school food policy each year. You can provide a rough estimation if you do not have a precise figure.

Subsidising school food

51. Beyond free school meals, please tell us about the subsidies/discounts in relation to food you offer to any of the following groups.

Group	Subsidies/discounts offered (y/n)	Annual cost of provision of these subsidies/discounts
Year 7 pupils		

Families with more than one child at school		
Children purchasing school lunch every day		
Staff		
Other group (please describe):		

52. Please tell us about any other subsidies / funding you offer to support pupils or staff to access food at school (including breakfast or an evening meal).

Other funding to support healthy eating

53. Please tell us about any other funding sources that support school food provision or food education (including funding source, value of funding and how this is spent).

Independent monitoring of school food

54. Have you sought expert and/or independent support for school food monitoring?

☐ Yes (please go to question 55)

☐ No (please go to question 57)

55. If yes, please provide an estimate of annual costs associated with school food monitoring.

£

56. Have you sought expert and/or independent support for any of the following tasks?

	Y / N
Drafting the catering contract	
Monitoring compliance with the School Food Standards	

Monitoring compliance with the Government Buying Standards	
Improving school food quality and take-up	
Teaching pupils about healthy eating or food preparation e.g. chefs	
Other (please specify):	

Training for school staff on health and well-being

57. In a typical year, do your staff undertake any training on health and well-being? This applies to teaching staff and any other school staff, including catering staff that are directly employed by the school (but not catering staff who are employed by external catering contractors). This may include internal or external workshops, qualifications/certificates, online training, webinars and attendance at conferences. Some examples may include the British Nutrition Foundation webinars, LACA seminars etc. ☐ Yes (please go to question 58)
☐ No (please go to question 59)

58. If yes, what type of health and well-being training is undertaken by staff in a typical school year? You can provide a rough estimate of cost if you do not have a precise figure.

Description of training	No. sessions	Total annual cost	Classroom cover required? (y/n)	Number of staff attending	Who covers the cost?
Workshop –external provider		£			
Workshop –in-house		£			
Course –external provider		£			
Course –in-house		£			
Formal qualification / certification		£			
Online training course		£			
Webinar		£			
Conference		£			
Other (please specify):		£			

If you are unable to complete this section, please ask the appropriate member of staff to complete it. Alternatively, you can provide us with the name/job title (and email address if you have it) of the appropriate staff member, and we will we contact them to complete it:

Other costs

59. Has your school incurred any of the costs associated with provision of food listed below?

Type of cost	Applicable (y/n)	Estimated annual spend	Who covers the cost?
Provision of water to pupils (e.g. water fountains)		£	☐ The school<br>☐ The catering provider<br>☐ Other (please specify):
School food clubs in which food is served (e.g. breakfast clubs, after school clubs)		£	☐ The school<br>☐ The catering provider<br>☐ Other (please specify):
Food rewards for good behaviour		£	☐ The school<br>☐ The catering provider<br>☐ Other (please specify):

Thank you for taking the time to complete the questionnaire

If there is anything additional you would like to tell us in relation to school food, please do so by writing in the space below. If you have nothing further to add please leave the space blank.
